# Modified Hongteng Baijiang decoction enema improves sequelae of pelvic inflammatory disease by regulating the LIF/JAK2/STAT3 pathway and gut microbiota

**DOI:** 10.1002/iid3.1300

**Published:** 2024-06-19

**Authors:** Xiaoli Ji, Quan Hu, Chengcheng Yang, Li Huang, Yefang Huang, Linwen Deng, Xiaoqing Song, Yongqing Zhang, Yan Wang

**Affiliations:** ^1^ Department of Gynecology Hospital of Chengdu University of Traditional Chinese Medicine Chengdu Sichuan China; ^2^ Department of Geriatrics Hospital of Chengdu University of Traditional Chinese Medicine Chengdu Sichuan China

**Keywords:** endometrial receptivity, gut microbiota, LIF/JAK2/STAT3 signaling pathway, modified Hongteng Baijiang decoction, sequelae of pelvic inflammatory disease

## Abstract

**Objective:**

The sequelae of pelvic inflammatory disease (SPID) are major causes of secondary infertility. Modified Hongteng Baijiang decoction (MHTBD) has produced positive results in the treatment of patients with chronic pelvic inflammatory disease; however, its role in SPID remains elusive. Therefore, this study clarified the role of MHTBD in SPID pathogenesis.

**Methods:**

The main components in MHTBD were analyzed by using liquid chromatography‒mass spectrometry (LC/MS). An SPID rat model was established, and the rats were treated with different doses of MHTBD (0.504 g of raw drug/kg, 1.008 g of raw drug/kg, and 2.016 g of raw drug/kg). Endometrial pinopodes were observed via scanning electron microscopy, endometrial thickness and inflammatory cell infiltration were assessed via HE staining, and the expression of estrogen receptor (ER), progesterone receptor (PR), integrin β3 (ITGB3), and CD31 in the endometrium was detected by using immunohistochemistry. Western blot analysis was used to detect the protein expression of LIF, JAK2, p‐JAK2, STAT3, and p‐STAT3 in the endometrium. Moreover, the changes in the gut microbiota were analyzed via 16S rRNA sequencing.

**Results:**

MHTBD improved endometrial receptivity, attenuated endometrial pathologic damage, reduced inflammatory cell infiltration, decreased ER and PR expression in the endometrium, and promoted the expression of LIF, p‐JAK2, and p‐STAT3 in the endometrium (*p* < .05) in SPID rats. Additionally, MHTBD treatment affected the composition of the gut microbiota in SPID rats. Furthermore, MHTBD attenuated endometrial receptivity and pathological damage in SPID rats by promoting the LIF/JAK2/STAT3 pathway.

**Conclusion:**

MHTBD attenuates SPID in rats by promoting the LIF/JAK2/STAT3 pathway and improving the composition of the gut microbiota. MHTBD may be a valuable drug for SPID therapy.

## Graphical abstract

The mechanism by which modified Hongteng Baijiang decoction improves endometrial receptivity in sequelae of pelvic inflammatory disease rats may be related to the LIF/JAK2/STAT3 signaling pathway and gut microbiota.

## 1 INTRODUCTION

Chronic endometritis (CE) is a condition in which the endometrium is infected and the inflammatory response persists; this disease can be classified as sequelae of pelvic inflammatory disease (SPID).^1^ Infertility, which is a common and frequent clinical disease, affects 14.5% of people of reproductive age, with secondary infertility accounting for approximately 2/3 of the cases.^2^ Tubal obstruction and pelvic adhesions caused by SPID are the primary factors of secondary infertility, and endometrial inflammation, uterine adhesions, and endometrial polyps caused by SPID also have important effects on pregnancy and pregnancy outcomes.^3^ Studies have shown that the incidence of CE in infertile patients is 2.8%−56.8%, and the incidence is as high as 67.6% and 56.8% in patients with recurrent spontaneous abortion (RSA) and recurrent implantation failure (RIF) in assisted reproduction, respectively.^4^, ^5^ These results show that CE is a common cause of both infertility and RSA and RIF in women, and infertility due to CE seriously affects women's reproductive health and quality of life.^6^ Therefore, active prevention and treatment of SPID are of great clinical and social significance for improving the pregnancy success rate of patients with CE infertility.

Currently, Western medicine interventions for CE infertility mainly involve antibiotic treatment, uterine cavity perfusion therapy, and light invasive endometrial surgery; however, the clinical efficacy is not satisfactory.^7^, ^8^ We conducted a preliminary study on the clinical efficacy and mechanism of action of the comprehensive treatment plan (Chinese medicine internal + Chinese medicine enema) and confirmed its efficacy and preliminary targets of action in alleviating clinical symptoms and improving pelvic signs and Chinese medicine symptoms.^9^ In addition, combined internal and external treatment with traditional Chinese medicine (TCM) via multiple routes of drug delivery has better clinical efficacy in treating infertility caused by CE, thus reflecting the advantages and characteristics of TCM.^10^, ^11^ Therefore, TCM may have certain efficacy in the treatment of SPID and warrants further study. Modified hongteng baijiang decoction (MHTBD) is a traditional formula consisting of Hongteng (*Sargentgloryvine Stem*), Baijiang (*Patrinia scabiosaefolia*), Dansheng (*Salvia miltiorrhiza Bge*), Dandelion (*Taraxacum mongolicum* Hand.‐Mazz.), Coix seed (*Coix lacryma‐jobi L. var. mayuen [Roman.] Stapf*), Chishao (*Paeonia lactiflora Pall*.), Yazhicao (*Commelina communis* L.), Plantain (*Plantago asiatica* L.), Sanleng (*Sparganium stoloni erum*, Buch.‐Ham.), Danpi (*Paeonia suffruticosa* Andr.), and Ezhu (*Curcuma phaeocaulis* Valeton), and most of these herbs have antioxidant, anti‐inflammatory, and antibacterial properties.^12^ Studies have indicated that MHTBD is clinically effective in treating patients with chronic pelvic inflammatory disease; moreover, it promotes effective relief of clinical symptoms and local signs and is safe to administer.^13^ However, the molecular mechanisms underlying the effect of MHTBD on SPID and its function are still widely unknown.

The gut microbiota is a microbial barrier against pathogen invasion, and studies have shown that the gut microbiota not only plays an important role in intestinal physiology but can also lead to the development and progression of various diseases, such as diabetes, multiple sclerosis, polycystic ovary syndrome, and endometriosis.^14^, ^15^ The gut microbiota has emerged as a new therapeutic target for diseases^16^; however, few data are available to explore the changes in gut microbes in individuals with SPID. Additionally, numerous basic and clinical studies have confirmed that LIF is a significant marker of endometrial receptivity and is closely related to embryo implantation; additionally, the LIF‐mediated JAK2‐STAT3 signaling pathway plays an important role in the development of embryo implantation.^17^, ^18^ CE can lead to a decrease in the level of LIF, thus causing abnormalities in the JAK2‐STAT3 signaling pathway, affecting endometrial receptivity, and ultimately leading to embryo implantation failure, which is a possible pathogenesis of CE infertility.^19^

Hence, this study aimed to elucidate whether MHTBD can alleviate the progression of SPID, including endometrial receptivity, tissue damage, and intestinal microbial changes, in a SPID model, as well as to explore whether the LIF/JAK2/STAT3 signaling pathway mediates this regulatory process. In this study, we treated SPID model rats with low, medium, or high doses of MHTBD and compared changes in endometrial receptivity, endometrial pathological changes, factors related to the LIF/JAK2/STAT3 signaling pathway, and the gut microbiota among the different groups to elucidate the mechanism by which MHTBD ameliorates SPID.

## 2 MATERIALS AND METHODS

### 2.1 Liquid chromatography‒mass spectrometry (LC/MS) analysis of MHTBD

One gram of the MHTBD sample was collected, 3 mL of 50% methanol was added, the sample was extracted via ultrasonication for 30 min, the sample was filtered through a 0.22 μm filter membrane, and 10 μL of the supernatant was collected for the assay. High‐performance LC (LC‐30; Shimadzu) coupled with a mass spectrometer (SCIEX 5600; AB SCIEX™) was used for analysis. The utilized chromatographic column was a Waters BEH C18 (150 × 2.1 mm, 2.5 µm) at 40°C with a flow rate of 0.3 mL/min. Mobile phase A was 0.1% HCOOH‐H_2_O, and mobile phase B was acetonitrile. Electrospray ionization (ESI)‐positive and negative ion modes were used for detection. The ESI source conditions were as follows: ion source gas 1 (Gas 1), 50; ion source gas 2 (Gas 2), 50; curtain gas (CUR), 25; source temperature, 500°C (positive ions) and 450°C (negative ions); ion secondary voltage floating, 5500 V (positive ions) and 4400 V (negative ions); TOF MS scan range, 100−1200 Da; product ion scan range, 50−1200 Da; TOF MS scan accumulation time, 0.2 s; product ion scan accumulation time, 0.2 s; and product ion scan accumulation time, 0.01 s. Secondary mass spectra were obtained by using information‐dependent acquisition in high‐sensitivity mode with a declustering potential of ±60 V and a collision energy of 35 ± 15 eV.

### 2.2 Establishment of the SPID model

Sixty female SPF‐rated SD rats that had reached sexual maturity but had not conceived (Chengdu University of Traditional Chinese Medicine; No. SCXK [Chuan] 2019‐11) weighing 220 ± 20 g were used. All of the rats were fed and watered freely, and ventilation and other conditions were under animal management regulations and approved by the Animal Ethics Committee of Chengdu University of Traditional Chinese Medicine (2022DL‐008). One week after adaptive feeding, 12 rats were randomly selected as the sham‐operated group (Sham), and the remaining rats were injected with a mixed bacterial solution (together with the mechanical injury method) to establish the SPID model.^20^ Briefly, the rats were anesthetized via intraperitoneal injection of sodium pentobarbital (35 mg/kg), and their limbs and heads were fixed in the supine position on a rat board. The rat's “Y‐shaped” uterus was exposed by taking a midline abdominal approach, cutting the abdominal length by approximately 1 cm with tissue scissors, inserting the needle from the bifurcation of the rat's “Y‐shaped” uterus, pulling it four times with a syringe in the direction of the ovaries, damaging the endometrial tissue on both sides and injecting 0.1 mL of mixed bacterial solution (6 × 10^7^/mL, 2:1 mixture of *E. coli* and *S. aureus*; ATCC) into each uterine cavity. After the operation was completed, the needle hole was closed with ophthalmic forceps, the uterus was retracted into the abdominal cavity, the incision was closed with sutures, and the incision was disinfected again. In the Sham group, the bacterial solution was replaced with sterile saline, and the rest of the operation was performed as described above. The rats were routinely maintained for 14 days, and five rats were randomly selected for pathological section observation to determine whether the model was successful.^21^

### 2.3 Animal grouping

After successful modeling, the model rats were randomly divided into the model group (SPID, saline), the low‐dose MHTBD group (SPID + MHTBD‐L, 0.504 g raw drug/kg), the medium‐dose MHTBD group (SPID + MHTBD‐M, 1.008 g raw drug/kg), the high‐dose MHTBD group (SPID + MHTBD‐H, 2.016 g raw drug/kg), the LIF inhibitor (EC330) group (SPID + EC330, 1 mg/kg), and the high‐dose MHTBD + EC330 group (SPID + MHTBD‐H + EC330, 2.016 g raw drug/kg + 1 mg/kg), with 12 rats in each group. For administration of the drugs, the rats were fasted for approximately 16 h before enema treatment, and cotton swabs were used to stimulate the anus of the rats to assist defecation before drug administration. The drug in the water bath was heated to 38−40°C, and the drug suspension (containing 0.504 g of raw drug/kg, 1.008 g of raw drug/kg, or 2.016 g of raw drug/kg for low, medium, or high levels, respectively) was administered by enema by using a syringe at a dose of 1 mL/100 g body weight. The enema was administered with a 16‐gauge gavage needle inserted to a depth of approximately 6−8 cm in the anus, and the drug was slowly injected. After the injection was completed, the rat's tail was lifted to keep the rat upside down for approximately 1 min, to allow the drug to flow deep into the intestinal cavity to prevent the drug from overflowing. Fasting ended at 2 h after the enema. All of the groups were given enemas once a day for 21 days. EC330 was injected intraperitoneally at 1 mg/kg once a day for 21 days. The utilized MHTBDs were Hongteng (20 g), Baijiang (20 g), Dansheng (20 g), dandelion (20 g), Coix seed (20 g), Chishao (15 g), Yazhicao (15 g), Plantain (15 g), Sanleng (10 g), Danpi (10 g), and Ezhu (10 g) (Chengdu University of Traditional Chinese Medicine). The above‐mentioned drugs were decocted three times and concentrated to the corresponding concentrations for administration.

### 2.4 Sample collection

After 21 days of drug administration, cecal fecal samples were collected from six rats in each group, and the endometrium was dissected and separated. The endometrial tissue on one side was fixed in 4% paraformaldehyde for pathological sectioning and immunohistochemistry, whereas the endometrial tissue on the other side was stored at −80°C for western blot analysis and qRT‐PCR detection. Subsequently, the remaining six rats in each group were routinely fed for 2 weeks, after which the rats were mated in a combined cage at a female:male ratio of 2:1. The female rats were observed to have vaginal plugs shed from their vaginal orifices (i.e., milky white solids at the vaginal orifices) on the following morning, and their vaginal smears showed a large amount of sperm, which was considered as the first day of pregnancy. On the fifth day after the confirmation of pregnancy, the rats were killed, and the pregnant uterus of the rats was dissected to observe the number of blastocysts. The endometrial tissue was fixed in 1% osmium acid for 2 h for scanning electron microscopy (SEM), to observe the pinopode density.

### 2.5 SEM

The endometrial tissue was removed after fixation with 1% osmium acid, washed twice with ultrapure water for 5 min each time, and dehydrated with a series of gradients of alcohol (30%, 50%, 70%, 80%, 90%, 95%, and 100%) for 10 min each. The samples were gently glued on conductive adhesive, ion sputtered, and sprayed (E‐1045; HITACHI). Finally, the images were observed and acquired via SEM (Inspect; FEI).

### 2.6 Hematoxylin−eosin (H&E) staining

The endometrial tissues were fixed in 4% paraformaldehyde, dehydrated in ethanol step by step, cleared in xylene for 40 min, embedded in paraffin, cut into 4−5 μm thick sections, and stained with H&E (cat. no. GP1031; Servicebio). The endometrial thickness and endometrial histopathology were observed under a microscope (BA210Digital; Motic). The inflammatory cell infiltration score was also calculated as follows: 0 points, no inflammatory cell infiltration; 1 point, a few scattered inflammatory cells within the lamina propria of the mucosa; 2 points, scattered focal but deep into the muscle with a small amount of inflammatory cell infiltration; and 3 points, most scattered or laminar inflammatory cells infiltrating and involving the whole layer, with a large amount of inflammatory cell infiltration.

### 2.7 Immunohistochemical staining

Endometrial tissues were taken, paraffin‐embedded, sectioned (3 μm), placed in citrate buffer for antigen repair, and incubated with estrogen receptor (ER, 1:200, cat. no. ab32063), progesterone receptor (PR, 1:200, cat. no. ab101688), integrin β3 (ITGB3, 1:200, cat. no. ab179473), and CD31 antibody (1:200, cat. no. ab28364) (Abcam) overnight at 4°C. The secondary antibody (cat. no. ab288151, 1:1000; Abcam) was added dropwise and incubated for 30 min at 37°C. DAB (K135925C; Beijing Zhongshirt Jinqiao Biological) was used for color development and observation under a microscope (BA210Digital; Motic). The immunohistochemically stained sections were analyzed by using an Image‐Pro Plus 6.0 image analysis system (Media Cybernetics).

### 2.8 Western blot analysis

The endometrial tissues were homogenized in prechilled RIPA lysis buffer (cat. no. P0013B; Beyotime) to extract total protein, and protein quantification was performed by using a BCA kit (cat. no. P0009; Beyotime). Electrophoresis was performed on 12% sodium dodecyl sulfate‒polyacrylamide gels and transferred to PVDF membranes, which were blocked with 5% BSA in TBST for 60 min. Primary antibodies against LIF (cat. no. A1288, 1:1000), JAK2 (cat. no. A7694, 1:1000), p‐JAK2 (cat. no. AP0531, 1:1000), STAT3 (cat. no. A1192, 1:1000), p‐STAT3 (cat. no. AP0715, 1:1000), and the internal reference β‐actin (cat. no. AC004, 1:2000) (ABclonal) were added, and the membranes were incubated overnight at 4°C. A horseradish peroxidase‐labeled secondary antibody (cat. no. ab288151, 1:8000; Abcam) was added, and the membrane was incubated at room temperature for 60 min. The membrane was subsequently washed three times with TBST for 15 min each time and finally developed via chemiluminescence (Affinity). The image information was collected on a gel imaging system (Tanon 5200), and the grayscale values of the protein bands were analyzed via ImageJ software by using β‐actin as the internal reference.

### 2.9 DNA extraction and 16S sequencing

Fecal DNA was extracted from the cecal fecal samples of each group of rats, and 16S rRNA sequencing and quantitative polymerase chain reaction (qPCR) of bacteria were performed to analyze the diversity of the fecal flora.

### 2.10 Statistical analysis

SPSS 20.0 software (IBM) was used for the statistical analysis. The data are expressed as the mean ± standard deviation (SD). Comparisons of data among groups were performed via one‐way ANOVA, followed by least significant difference tests. A *p* value < .05 was considered to be statistically significant.

## 3 RESULTS

### 3.1 Analysis of the active ingredients of MHTBD

**Figure 1:**
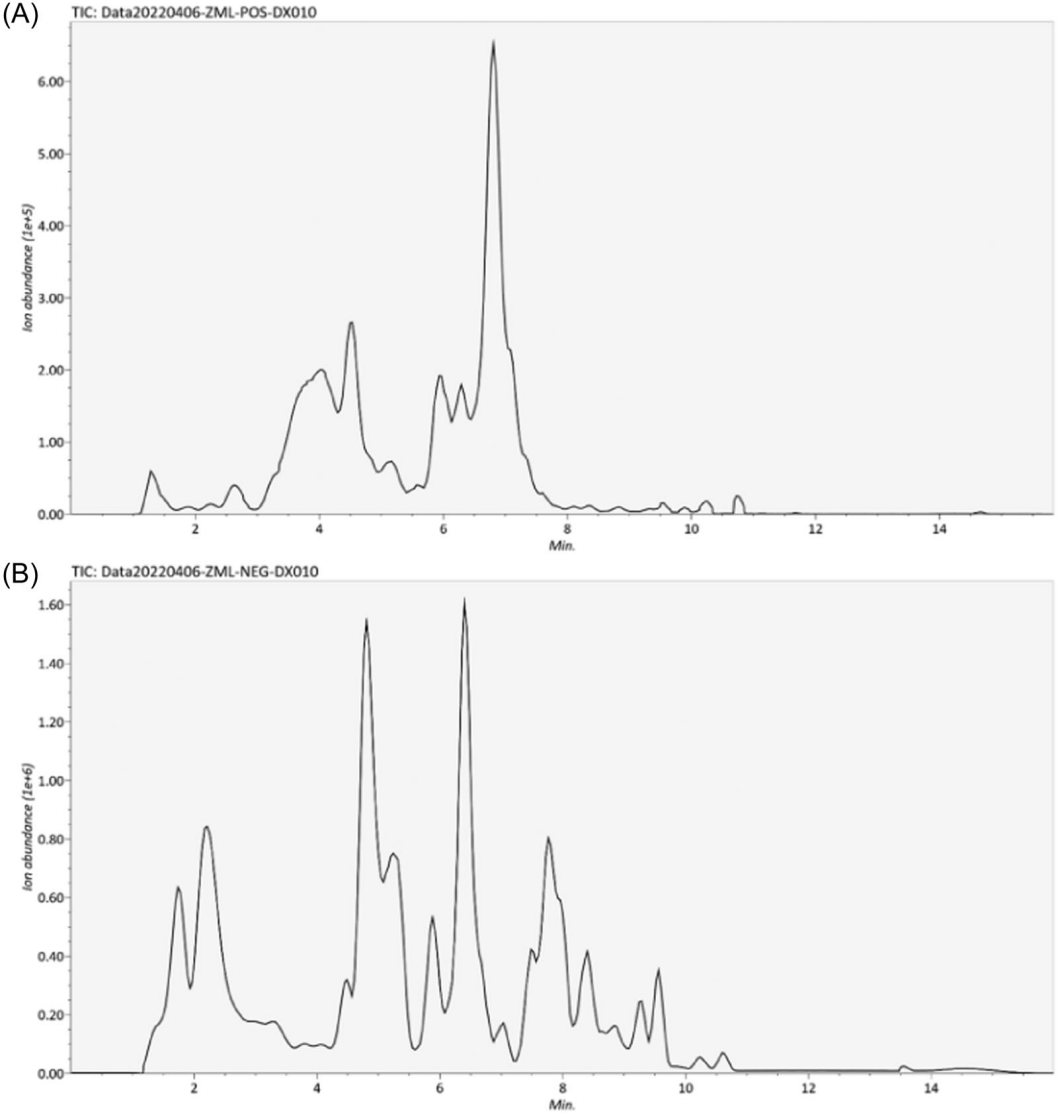
Analysis of the active ingredients of MHTBD. The chromatograms of positive ions (A) and negative ions (B) of the MHTBD sample. MHTBD, modified Hongteng Baijiang decoction.

Figure 1 shows chromatograms of the positive (Figure 1A) and negative (Figure 1B) ions of MHTBD. The raw data obtained via LC‒MS were converted by using Analysis Base File Converter software, and the converted formatted raw data were imported into MS‐DIAL 4.60 software for preprocessing, including peak extraction, noise removal, deconvolution, and peak alignment. The extracted peak information was compared with the MassBank, Respect, and GNPS databases. The details of the active ingredients of MHTBD determined via positive and negative ion chromatography are listed in Table S1. The results in Table S1 demonstrate that among the active ingredients of MHTBD, those with the highest peak areas were 3‐feruloylquinic acid (C_17_H_20_O_9_), caffeoyl quinic acid (C_16_H_18_O_9_), 2‐(hydroxymethyl)−4(3H)‐quinazolinone (C_9_H_8_N_2_O_2_), and circumdatin F_130023 (C_17_H_13_N_3_O_2_). The chemical information of the MHTBD was collected as a whole, and 377 incoming chemical components were initially identified, thus suggesting the pharmacodynamic basis of the MHTBD.

### 3.2 Effect of MHTBD enemas on endometrial pathology

**Figure 2:**
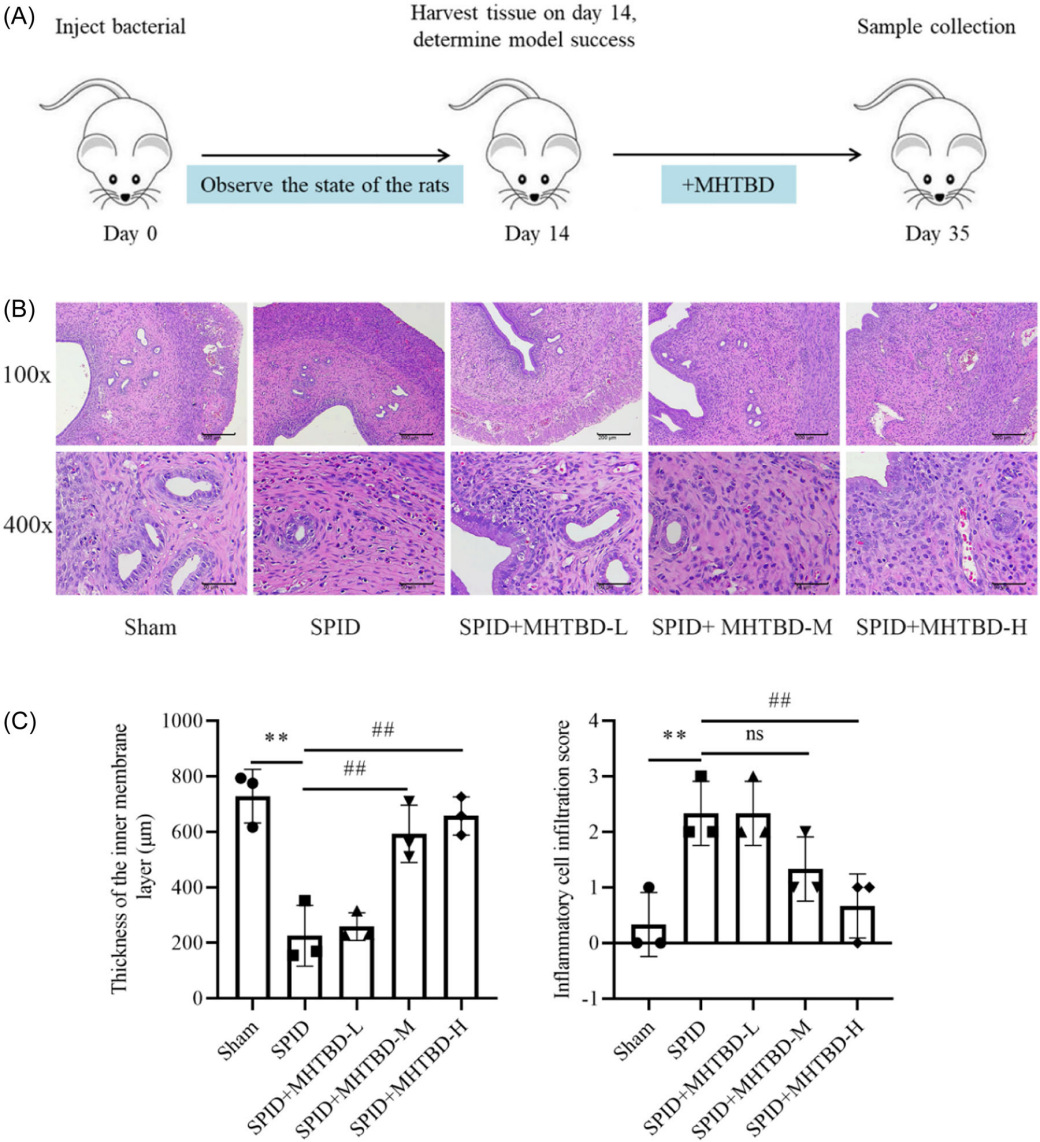
Effect of MHTBD enemas on endometrial pathology. Schematic diagram of the experiment (A). Endometrial tissue was observed via H&E staining (magnification, ×100 and ×400; scale bars, 200 and 50 μm; *n* = 3) (B). Thickness of the inner membrane layer and inflammatory infiltration score (C). Compared with the Sham group, **p < .01; compared with the SPID group, ^##^ *p* < .01, ^ns^ *p* > .05. H&E, hematoxylin−eosin; MHTBD, modified Hongteng Baijiang decoction; SPID, sequelae of pelvic inflammatory disease.

The general experimental protocol was as follows (Figure 2A). To confirm the effect of MHTBD enemas on endometrial pathology, we performed H&E staining. The results indicated thinning of the endometrial layer, more degenerative necrosis of epithelial cells and a high amount of inflammatory cell infiltration in the lamina propria in the SPID rats. However, in the SPID + MHTBD‐H group, the endometrial layer was slightly thinner, and the lamina propria was infiltrated by a small number of inflammatory cells. Compared with that in the SPID group, the endometrial thickness in the SPID + MHTBD‐H group was significantly greater, whereas the inflammatory cell infiltration score was significantly lower (*p* < .01, Figure 2B,C), thus suggesting that MHTBD attenuates pathological damage in SPID patients.

### 3.3 Effect of MHTBD enema on the receptivity of the rat endometrium

**Figure 3:**
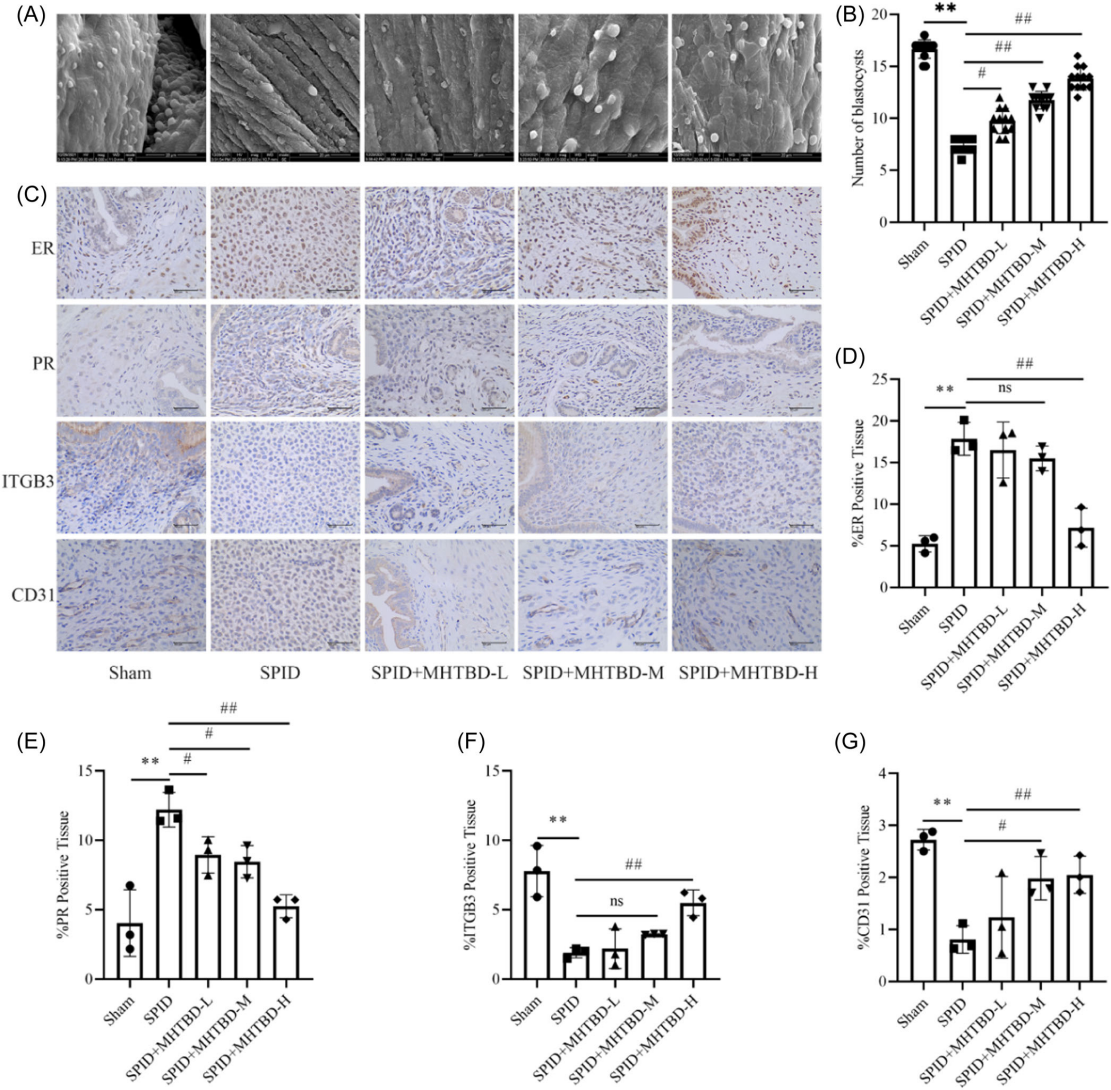
Effect of MHTBD enema on the receptivity of the rat endometrium. Scanning electron microscopy (magnification, ×5000, scale bar, 20 μm) (A). Number of blastocysts (*n* = 12) (B). Immunohistochemical staining of ER, PR, ITGB3, and CD31 in endometrial tissue (magnification, ×40; scale bar, 50 μm; *n* = 3) (C). Positive expression of ER (D), PR (E), ITGB3 (F), and CD31 (G) in endometrial tissue (*n* = 3). The data are expressed as the mean ± SD. Compared with the Sham group, **p < .01; compared with the SPID group, ^#^ *p* < .05, ^##^ *p* < .01; ^ns^ *p* > .05. ER, estrogen receptor; ITGB3, integrin β3; MHTBD, modified Hongteng Baijiang decoction; SPID, sequelae of pelvic inflammatory disease.

As shown in Figure 3A,B, the endometrial pinopode was observed by using SEM to evaluate the maturation of the endometrial pinopode and to count the blastocysts. The results showed that there were a large number of “mushroom‐shaped” protrusions on the surface of the endometrial epithelial cell membrane in the sham group; these protrusions were well defined, lacked microvilli and exhibited fully developed pinopodes. In the SPID group, the membrane surface of the endometrial epithelial cells was heterogeneous, with a small number of pinopodes focally expressed, and most of the endometrial surface was free of membranous protrusions and covered with dense microvilli. In the SPID + MHTBD‐L group, a small number of pinopodes, which are developmental pinopodes, were locally expressed in the endometrium. In the SPID + MHTBD‐M group, a large number of pinopodes were expressed on the endometrial surface, but there were still a few short and thin microvilli. The morphology of the endometrial epithelial pinopodes in the SPID + MHTBD‐H group was relatively uniform in size; moreover, development was basically synchronized, and there was no significant difference in development between the SPID + MHTBD‐H and Sham groups. In addition, the number of blastocysts was significantly lower in the SPID group than in the Sham group, and MHTBD treatment significantly increased the number of blastocysts compared with that in the SPID group (*p* < .05), thus indicating that MHTBD treatment increased the number of blastocysts and improved the maturation of pinopodes in SPID rats. Immunohistochemical staining demonstrated that the expression of ER and PR was markedly greater and the expression of ITGB3 and CD31 was noticeably lower in the endometria of the SPID group than in those of the Sham group, and MHTBD‐H treatment reversed these changes (*p* < .05, Figure 3C−G). These data suggest that MHTBD can inhibit SPID progression.

### 3.4 Effect of MHTBD enemas on the endometrial LIF/JAK2/STAT3 signaling pathway

**Figure 4:**
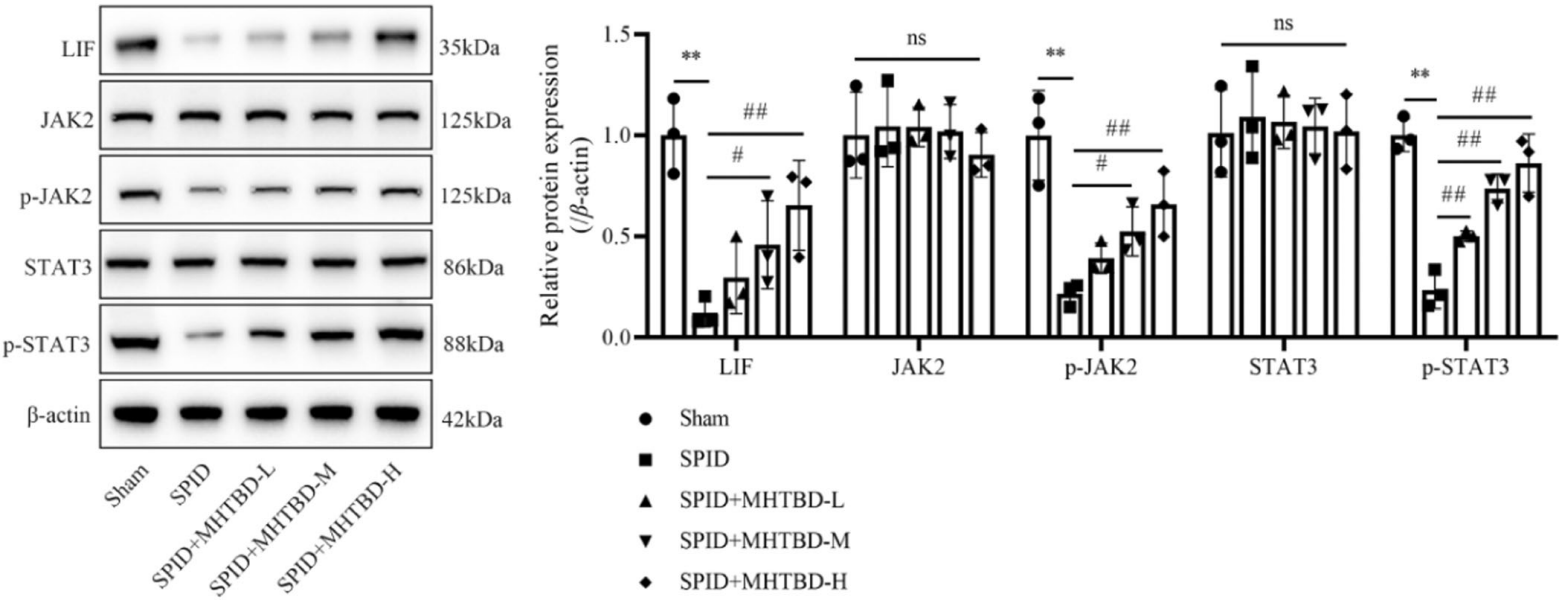
Effect of MHTBD enemas on the endometrial LIF/JAK2/STAT3 signaling pathway. Relative protein expression of LIF, JAK2, p‐JAK2, STAT3, and p‐STAT3 in endometrial tissue (*n* = 3); protein bands were calculated as a ratio relative to β‐actin protein levels. The data are expressed as the mean ± SD. Compared with the Sham group, **p < .01; compared with the SPID group, ^#^ *p* < .05, ^##^ *p* < .01; ^ns^ *p* > .05. MHTBD, modified Hongteng Baijiang decoction; SPID, sequelae of pelvic inflammatory disease.

Subsequently, the effect of MHTBD enemas on the endometrial LIF/JAK2/STAT3 signaling pathway was assessed. The results showed that the protein expression of LIF, p‐JAK2, and p‐STAT3 was significantly lower in the endometrial tissue of the SPID group than in that of the Sham group (*p* < .01). In contrast, MHTBD‐M and MHTBD‐H treatment rescued these effects (*p* < .05, Figure 4), thus suggesting that MHTBD promotes the LIF/JAK2/STAT3 signaling pathway in SPID.

### 3.5 Effect of the MHTBD‐mediated LIF/JAK2/STAT3 signaling pathway on endometrial pathology and receptivity in rats

**Figure 5:**
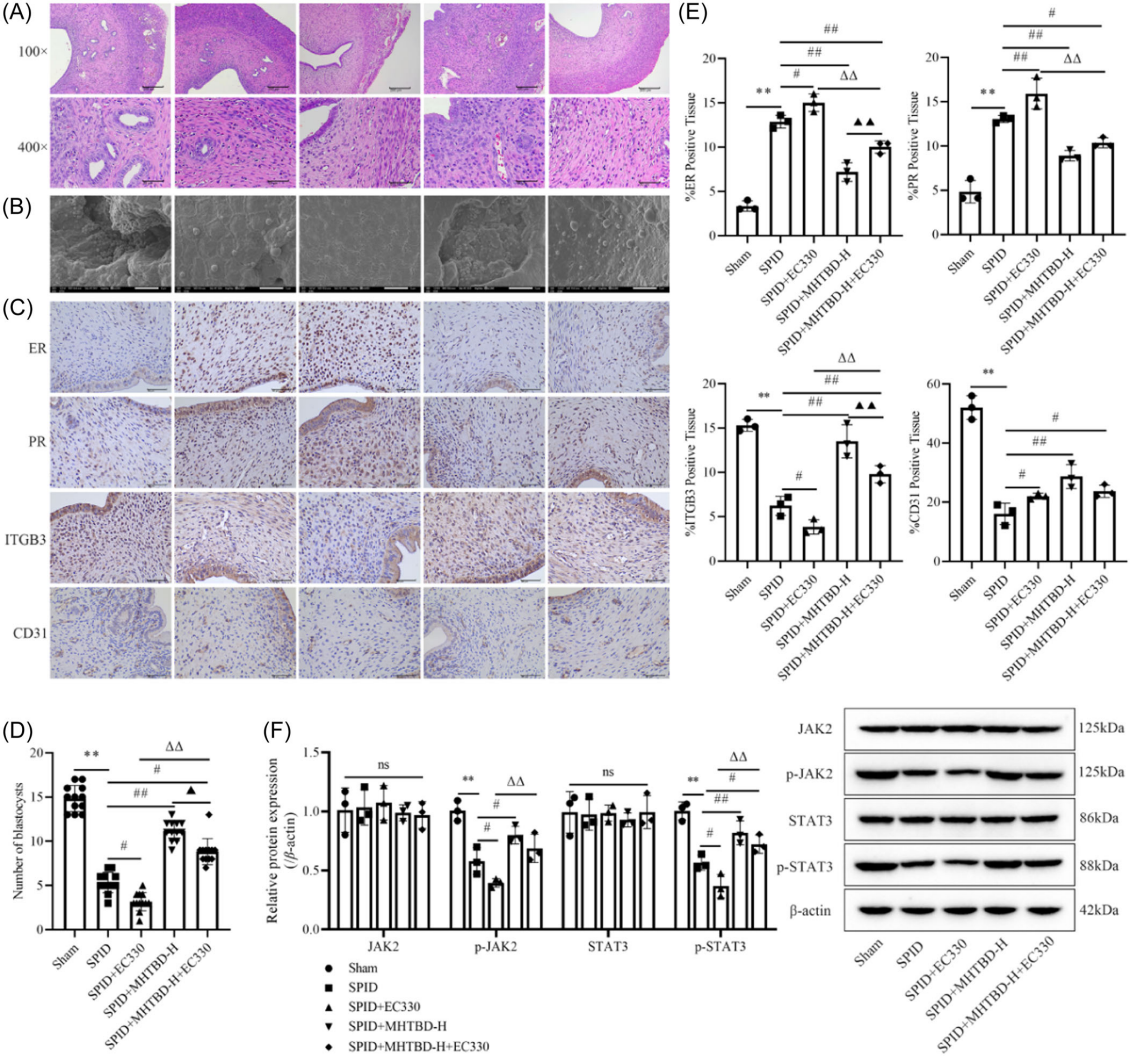
Effect of the MHTBD‐mediated LIF/JAK2/STAT3 signaling pathway on endometrial pathology and receptivity in rats. Endometrial tissue was observed via H&E staining (magnification, ×100 and ×400; scale bars, 200 and 50 μm; *n* = 3) (A). Scanning electron microscopy (magnification, ×5000; scale bar, 5 μm) (B). Immunohistochemical staining of ER, PR, ITGB3, and CD31 in endometrial tissue (magnification, ×40; scale bar, 50 μm; *n* = 3) (C). Number of blastocysts (*n* = 12) (D). Positive expression of ER, PR, ITGB3, and CD31 in endometrial tissue (*n* = 3) (E). Relative protein expression of JAK2, p‐JAK2, STAT3, and p‐STAT3 in endometrial tissue (*n* = 3). The protein band density was calculated as the ratio relative to the β‐actin protein concentration (F). The data are expressed as the mean ± SD. Compared with the Sham group, **p < .01; compared with the SPID group, ^#^ *p* < .05, ^##^ *p* < .01; compared with the SPID + EC330 group, ^ΔΔ^ *p* < .01; compared with the SPID + MHTBD group, ^▲^ *p* < .05, ^▲▲^ *p* < .01; ^ns^ *p* > .05. ER, estrogen receptor; H&E, hematoxylin−eosin; ITGB3, integrin β3; MHTBD, modified Hongteng Baijiang decoction; SPID, sequelae of pelvic inflammatory disease.

To confirm the protective effect of MHTBD against SPID by promoting the LIF/JAK2/STAT3 pathway, we further treated SPID rats with a LIF inhibitor (EC330) or MHTBD + EC330. Compared with that in the SPID + EC330 group, the pathological damage to uterine histopathology was significantly reduced in the SPID + MHTBD + EC330 group (Figure 5A), a large number of pinopodes were densely distributed in the folds of the endometrium, and the number of blastocysts was significantly increased in the endometrium (*p* < .01, Figure 5B,D). In addition, compared with those in the SPID + EC330 group, the expression of ER and PR was significantly lower, and the expression of ITGB3 was significantly greater in the endometrium of the SPID + MHTBD + EC330 group (*p* < .01, Figure 5C,E). Western blot analysis demonstrated that the protein expression of p‐JAK2 and p‐STAT3 was significantly greater in the endometrium of the SPID + MHTBD + EC330 group than in that of the SPID + EC330 group (*p* < .05, Figure 5F). These results demonstrated that MHTBD protects against SPID by promoting the LIF/JAK2/STAT3 pathway.

### 3.6 Effect of MHTBD enema on the gut microbiota of SPID‐treated rats

**Figure 6:**
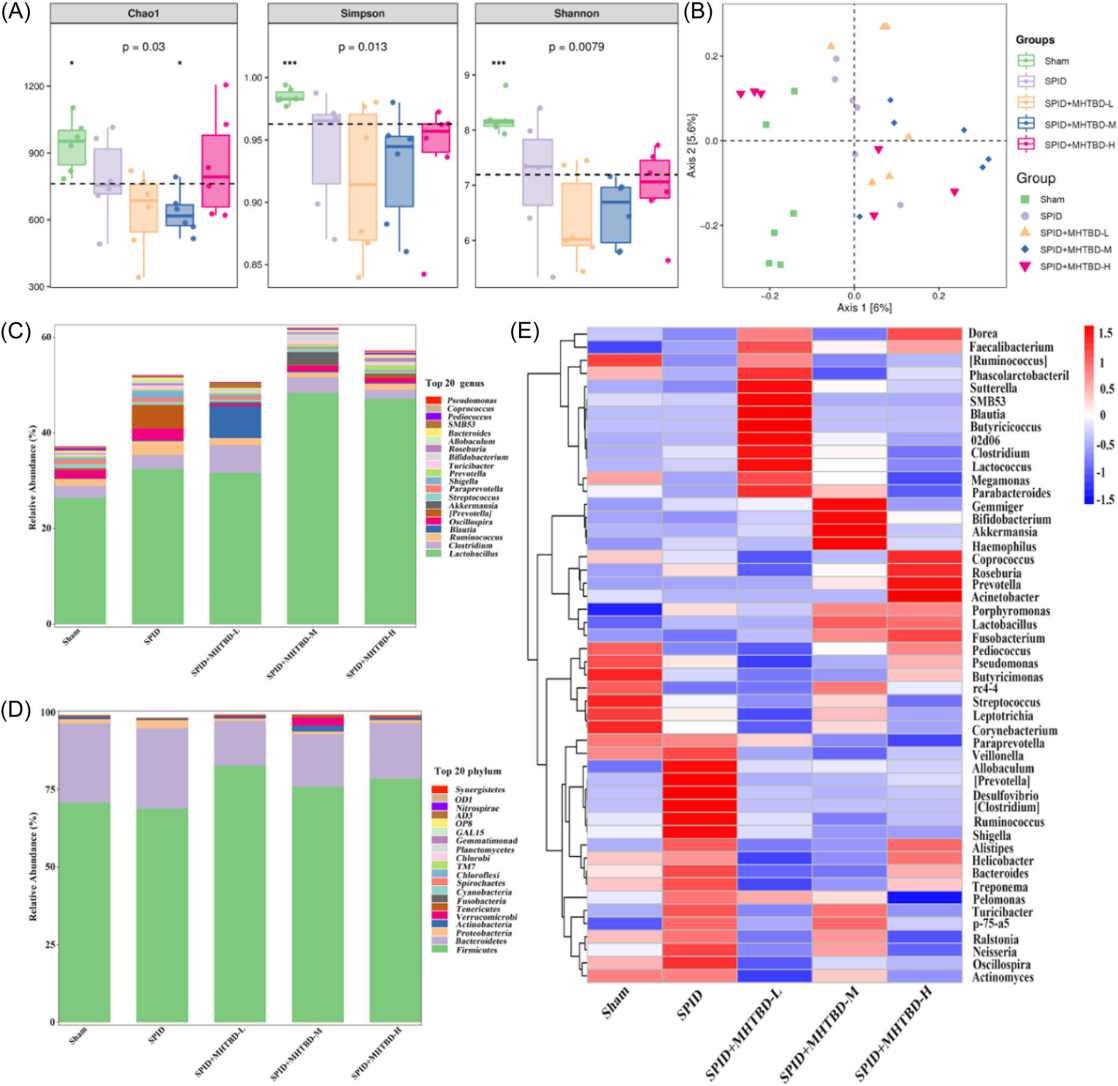
Effect of MHTBD enema on the gut microbiota of SPID rats. The diversity of the fecal microbiota in the rats (*n* = 6) (A). PCoA analysis of Bray‒Curtis distances in the PC1 and PC2 axes in rats (*n* = 6) (B). The relative abundance at the phylum level (*n* = 6) (C). The relative abundance at the genus level (*n* = 6) (D). Heatmap analysis of changes in the gut microbiota after different treatments (*n* = 6) (E). *p < .05, **p < .01, ***p < .001. MHTBD, modified Hongteng Baijiang decoction; SPID, sequelae of pelvic inflammatory disease.

16S sequencing was used to determine the effect of MHTBD enemas on the gut microbiota of rats. The results showed that the Chao1 index, Simpson index, and Shannon index were significantly lower in the SPID group than in the Sham group, whereas the SPID + MHTBD‐H group suppressed these changes (*p* < .05), thus demonstrating that MHTBD‐H may increase the abundance of microbiota in rats (Figure 6A). In addition, the spatial distributions of the gut microbiota in the SPID and Sham groups were significantly different, whereas the SPID + MHTBD‐H treatment promoted changes in the gut microbiota of the rats in different spatial directions (Figure 6B). These results confirmed that MHTBD treatment affected the gut microbiota composition of SPID rats.

To further explore the effect of MHTBD on the abundance of gut microbiota in SPID rats, the abundance of gut microbiota at the phylum and genus levels was analyzed. At the phylum level, the relative abundances of *Firmicutes*, *Bacteroidetes*, and *Proteobacteria*, among other phyla, were changed in the SPID group compared to those in the Sham group; however, MHTBD treatment reversed these changes (Figure 6C). At the genus level, the relative abundances of *Lactobacillus*, *Clostridium*, *Ruminococcus*, and *Oscillospira* were greater in the SPID group than in the Sham group. In SPID rats treated with MHTBD‐H, the relative abundance of *Lactobacillus* increased, whereas the relative abundances of *Clostridium*, *Ruminococcus*, and *Oscillospira* decreased (Figure 6D), thus demonstrating that MHTBD affected the abundance of the gut microbiota in SPID rats. A heatmap also demonstrated that the proportions of *Pediococcus*, *Pseudomonas*, *Butyricimonas*, *Streptococcus*, *Leptotrichia*, and *Corynebacterium* decreased and the proportions of *Paraprevotella*, *Veillonella*, *Allobaculum*, *Desulfovibrio*, *Ruminococcus*, *Shigella, Alistipes, Helicobacter, Bacteroides, Treponema, Pelomonas, Turicibacter, Oscillospira*, and *Actinomyces* increased in the SPID group compared with those in the Sham group. Compared with those in the Sham group, the proportions of *Paraprevotella*, *Veillonella*, *Allobaculum*, *Desulfovibrio*, *Ruminococcus*, *Shigella, Bacteroides, Treponema, Pelomonas, Turicibacter, Oscillospira*, and *Actinomyces* were reduced in the MHTBD group, and the therapeutic effect of MHTBD may be related to the differential expression of these gut microbiota (Figure 6E). In brief, we demonstrated that MHTBD affects the abundance of the gut microbiota in SPID rats.

## 4 DISCUSSION

In the present study, we demonstrated that MHTBD improved endometrial receptivity, alleviated endometrial lesions, and promoted the activation of the LIF/JAK2/STAT3 signaling pathway in the endometria of SPID rats. Further results showed that MHTBD affected the abundance of the gut microbiota in SPID rats. This finding suggested that the improvement of SPID by MHTBD may be achieved by modulating the gut microbiota and the LIF/JAK2/STAT3 signaling pathway.

The examination of the chemical composition of TCMs is a prerequisite for elucidating the pharmacodynamic substances, mechanism of action, and clinical efficacy of TCMs.^22^ However, due to the complexity of TCM composition, the effective components exerting pharmacodynamic effects are still unclear, which directly affects the assessment of quality control, drug metabolism, and active ingredient screening, among other factors.^23^ LC‒MS technology has played a driving role in the modernization of TCM.^24^ In this study, we preliminarily analyzed the active components of MHTBD and initially identified 377 incoming chemical components. The components with the greatest peak areas were 3‐feruloylquinic acid (C_17_H_20_O_9_), caffeoyl quinic acid (C_16_H_18_O_9_), 2‐(hydroxymethyl)−4(3H)‐quinazolinone (C_9_H_8_N_2_O_2_), and circumdatin F_130023 (C_17_H_13_N_3_O_2_). The present results provide important pharmacokinetic information for the further development and study of MHTBD; however, it is still necessary to further identify the main components involved in the treatment of MHTBD.

Rats are widely used in SPID experimental studies; specifically, bacterial modeling combined with mechanical injury can shorten the modeling time and increase the success rate of infection, and the pathological manifestations, inflammatory pathways, and lesion mechanisms are more similar to those of human SPID.^21^ In this study, the SPID rat model was established by using bacterial modeling combined with the mechanical injury method.^25^ After modeling, the endometrial layer of the rats was thinned, more epithelial cells were degenerated and necrotic, more inflammatory cells had infiltrated the lamina propria, and the inflammatory cell infiltration score of the uterus in the SPID group was greater than that in the Sham group, which was consistent with the observed pathological changes in the SPID group. After treatment with MHTBD, endometrial histopathological changes were significantly improved, and endometrial inflammatory cell infiltration was significantly reduced, thus indicating that MHTBD can reduce inflammatory cell infiltration in the rat uterus and has a therapeutic effect on SPID. In the future, the role of MHTBD in cellular and clinical settings needs to be further investigated.

Endometrial receptivity is a state in which the embryo can adhere to the endometrium, invade the endometrium, and undergo a series of changes in the endometrial mesenchyme.^26^ Endometrial receptivity is an important indicator of endometrial function because it is correlated with endometrial status and ovarian function.^27^ The pinopode reduces the volume of the uterine cavity, contributes to the positioning of the embryo in the endometrium, and prevents the removal of the embryo from the epithelial cell surface; moreover, a fully developed pinopode is a vital morphological marker for the establishment of endometrial receptivity and the opening of the implantation window.^28^ In the present study, we found that MHTBD had an upregulatory effect on the SPID‐induced decrease in pinopode maturation in a dose‐dependent manner, and MHTBD treatment increased the number of blastocysts, thus illustrating that MHTBD can enhance endometrial receptivity by improving the pinopode in SPID rats. Published studies have demonstrated that estrogen and progesterone are the primary hormones that guide the establishment of endometrial receptivity, whereas the expression of ER and PR directly determines the ability of the endometrium to respond to hormones.^29^ ITGB3 is expressed on the surface of the endometrium and is closely related to endometrial receptivity.^30^ CD31 is an endothelial cell marker that can more accurately reflect the number of blood vessels.^31^ Similar to previous findings,^32^ our results also showed that MHTBD inhibited the expression of ER and PR and promoted the expression of ITGB3 and CD31 in the endometria of SPID rats. These results demonstrated that MHTBD can regulate molecular markers of endometrial receptivity.

Studies have reported that leukemia inhibitory factor (LIF) and its JAK2/STAT3 signaling pathway are among the pathways most closely associated with infertility due to abnormal endometrial receptivity.^33^ LIF, which is a member of the interleukin‐6 family, activates the intracellular nonreceptor JAK by binding to the receptor LIFR, thus triggering the phosphorylation of tyrosine residues in the cytoplasm. The phosphorylated JAK site can recruit tyrosine residues at position 705 of the SH2 region of STAT3. The phosphorylated SH2 region of STAT3 then binds to each other to form SH2 dimers, which can subsequently be translocated to the nucleus, where the dimer STAT3 can activate downstream transcription factors that are important for follicle growth and development, embryo adhesion, and embryo implantation.^34^, ^35^ Several previous reports have demonstrated that CE decreases the level of LIF, thus causing the abnormal function of the JAK2/STAT3 signaling pathway, which affects endometrial receptivity and ultimately leads to embryo implantation failure and is a possible pathogenesis of CE infertility.^36^ In the current study, the results were the same as those described above, and MHTBD significantly upregulated the expression of LIF, p‐JAK2, and p‐STAT3 in SPID rats, thus suggesting that MHTBD may improve endometrial receptivity by regulating the LIF/JAK2/STAT3 signaling pathway. EC330 is an LIF inhibitor^37^; in this study, the application of EC330 promoted uterine histopathological injury and increased the expression of ER and PR in the endometrium, whereas MHTBD + EC330 significantly downregulated the expression of ER and PR in the endometrium and attenuated uterine histopathological injury. This finding suggested that MHTBD inhibits the development of SPID by promoting the LIF/JAK2/STAT3 pathway.

The human gut colonizes approximately 10^14^ bacteria, which is approximately 10 times the number of cells in the human body.^38^ Reports have demonstrated the presence of gut microbiota in the cervical canal of patients with chronic pelvic inflammatory disease, which is naturally resistant to a variety of antibiotics and affects the normal gut microbiota in the vagina.^39^ To further investigate the effect of MHTBD on the gut microbiota of SPID rats, we performed 16S rDNA high‐throughput sequencing analysis of the gut microbiota. The results of this study showed that bacterial diversity (alpha index) was reduced in SPID rats, and MHTBD treatment inhibited this change. Furthermore, the gut microbiota balance was significantly disrupted in the SPID rats, whereas MHTBD improved the abundance of the gut microbiota in the SPID rats, thus indicating that MHTBD helps to establish a relatively stable gut microbiota balance. Changes in the gut microbiota are associated with estrogen metabolism in vivo, and the abundance of *Firmicutes* is thought to be related to endometriosis^40^; moreover, women with endometriosis are more inclined to have *Shigella*/*Escherichia coli* as the dominant flora in the fecal microbiota.^41^ Yu et al.^42^ reported that β‐sitosterol could improve the gut microbiota in patients with polycystic ovary syndrome by reducing the abundance of *Firmicutes* and *Lactobacillus*. Our study showed that the abundances of *Bacteroidetes*, *Proteobacteria*, *Lactobacillus*, *Clostridium*, *Ruminococcus*, and *Oscillospira* increased in SPID rats, whereas MHTBD treatment inhibited the increase in the abundances of the above‐mentioned flora (but not *Lactobacillus*). *Lactobacillus* is known to be beneficial to human health because of its probiotic function^43^; in the present study, MHTBD increased the abundance of *Lactobacillus*. However, using heatmap analysis, we found that MHTBD treatment also affected the abundance of *Pediococcus*, *Pseudomonas*, *Butyricimonas*, *Streptococcus*, *Leptotrichia*, and *Corynebacterium*; however, we need to perform more studies in the future for validation of these results. In addition, dysbiosis of the gut microbiota releases more harmful metabolites, thus leading to an increase in harmful metabolites entering the circulation and triggering inflammatory signaling pathways.^44^ The LIF/JAK2/STAT3 pathway plays an important role in inflammation, and intestinal microbiota dysbiosis may be associated with the LIF/JAK2/STAT3 pathway.^45^ However, this study lacked research on the relationship between the LIF/JAK2/STAT3 signaling pathway and the gut microbiota, as well as metabolomics experiments. Future studies will help to gain insight into the relationship between LIF/JAK2/STAT3 and the gut microbiota and its effect on SPID.

In conclusion, we clarified that MHTBD improved endometrial receptivity, attenuated endometrial pathologic damage, decreased the inflammatory cell infiltration score, and promoted the LIF/JAK2/STAT3 signaling pathway in SPID rats and that MHTBD also affected the composition of the gut microbiota in SPID rats. In addition, MHTBD attenuated endometrial receptivity and pathological damage in SPID rats by promoting the LIF/JAK2/STAT3 pathway. MHTBD may be a valuable drug for the treatment of SPID. The findings of this study provide scientific evidence for the use of MHTBD in the treatment of SPID and offer insights for further exploration of its potential applications.

## AUTHOR CONTRIBUTIONS

Xiaoli Ji, Quan Hu, and Yan Wang conceived and designed the research. Xiaoli Ji, Quan Hu, Chengcheng Yang, Li Huang, Yefang Huang, and Linwen Deng performed most of the experiments and wrote the paper. Xiaoli Ji, Quan Hu, and Xiaoqing Song performed parts of the experiments. Xiaoli Ji and Yongqing Zhang analyzed the data. All of the authors contributed to the article and approved the submitted version.

## CONFLICT OF INTEREST STATEMENT

The authors declare no conflict of interest.

## ETHICS STATEMENT

This study was authorized by the Animal Ethics Committee of Chengdu University of Traditional Chinese Medicine (2022DL‐008).

## Supporting information


**Table S1**: Details of the active ingredients of MHTBD determined via positive and negative ion chromatography.

## Data Availability

The data that support the findings of this study are available from the corresponding author on reasonable request. After the publication of the study findings, the data will be available for others to request. The research team will provide an email address for communication once the data are approved to be shared with others. All of the data that were generated or used during this study are included in this published article, and further details are available upon request from the corresponding author.
