# Supplementary material for: Modified Hongteng Baijiang decoction enema improves sequelae of pelvic inflammatory disease by regulating the LIF/JAK2/STAT3 pathway and gut microbiota
Source: Immun Inflamm Dis. 2024 Jun 19;12(6):e1300. doi: 10.1002/iid3.1300 (PMC11186298; doi:10.1002/iid3.1300)
Supplement: Supplementary file 1 — Table S1: Details of the active ingredients of MHTBD determined via positive and negative ion chromatography. [file IID3-12-e1300-s001.pdf]

Title	RT (min)	Precursor m/z	Height	Area	Formula	Ontology	InChIKey	SMILES
Rosmarinic acid	6.199217	359.0748	206459.1875	2587157.5	C18H16O8	Coumaric acids and derivatives	DOUMFZQKYFQNTF-WUTVXBCWSA-N	OC(=O)[C@@H](CC1=CC(O)=C(O)C=C1)OC(=O)\C=C\C1=CC(O)=C(O)C=C1
3,4-di-O-caffeoylquinic acid	5.880567	515.1185	49265.3125	607199.9375	C25H24O12		UFCLZKMFXSILNL-RVXRWRFUSA-N	O=C(OC1CC(O)(C(=O)O)CC(O)C1OC(=O)C=CC2=CC=C(O)C(O)=C2)C=CC3=CC=C(O)C(O)=C3
Protocatechuic aldehyde	2.605567	137.0257	155768.8125	2752560	C7H6O3	Hydroxybenzaldehydes	IBGBGRVKPALMCQ-UHFFFAOYSA-N	OC1=C(O)C=C(C=O)C=C1
Berberine	6.8067	336.121	396343.3125	8536775	C20H18NO4	Protoberberine alkaloids and derivatives	YBHILYKTIRIUTE-UHFFFAOYSA-N	COC1=C(OC)C2=C[N+]3=C(C=C2C=C1)C1=CC2=C(OCO2)C=C1CC3
(2R)-2-[(E)-3-[3-[(1R)-1-carboxy-2-(3,4-dihydroxyphenyl)ethoxy]carbonyl-2-(3,4-dihydroxyphenyl)-7-hydroxy-2,3-dihydro-1-benzofuran-4-yl]prop-2-enoyl]oxy-3-(3,4-	6.039233	717.1433	122662.6875	1325543.625	C36H30O16	2-arylbenzofuran flavonoids	SNKFFCBZYFGCQN-FTNWOIIGSA-N	O=C(OC(C(=O)O)CC1=CC=C(O)C(O)=C1)C=CC2=CC=C(O)C=3OC(C4=CC=C(O)C(O)=C4)C(C(=O)OC(C(=O)O)CC5=CC=C(O)C(O)=C5)C23
Benzoic acid	7.800817	123.0428	3072.6875	36962.05078	C7H6O2	Benzoic acids	WPYMKLBDIGXBTP-UHFFFAOYSA-N	O=C(O)C=1C=CC=CC1
o-Phenanthroline	5.244617	181.074	3634.375	51766.98828	C12H8N2	Phenanthrolines	DGEZNRSVGBDHLK-UHFFFAOYSA-N	C1=CC2=C(N=C1)C1=C(C=CC=N1)C=C2
C27H46O14	10.35782	593.2697	5033.6875	103547.8906	C27H46O14	Formula predicted		
Sebacic acid	6.994667	201.1131	14729.5	184171.75	C10H18O4	Medium-chain fatty acids	CXMXRPHRNRROMY-UHFFFAOYSA-N	OC(=O)CCCCCCCCC(O)=O
2-(2-hydroxyphenyl)-4-phenylbenzene-1,3,5-triol	6.290067	336.121	175840.375	2415678.25	C18H14O4	M-terphenyls	UYKZGYUZJASKNB-UHFFFAOYSA-N	OC=1C=CC=CC1C2=C(O)C=C(O)C(=C2O)C=3C=CC=CC3
Caffeic acid	3.3227	179.0343	175551.5	5071756.5	C9H8O4	Hydroxycinnamic acids	QAIPRVGONGVQAS-UHFFFAOYSA-N	O=C(O)C=CC1=CC=C(O)C(O)=C1
[(1S,2S,3R,5R,6S,8S)-6-hydroxy-8-methyl-3-{[(2S,3R,4S,5S,6R)-3,4,5-trihydroxy-6-(hydroxymethyl)oxan-2-yl]oxy}-9,10-dioxatetracyclo[4.3.1.0?,?.0?,?]decan-2-yl]methyl benzoate	4.7283	503.1495	71482.625	1018877.625	C23H28O11	Terpene glycosides	YKRGDOXKVOZESV-RMNZJSDUSA-N	O=C(OCC12C3OC4(O)CC(O3)(C)C2(OC5OC(CO)C(O)C(O)C5O)CC41)C=6C=CC=CC6
2-[4-[(E)-2-carboxyethenyl]-2-(3,4-dihydroxyphenyl)-7-hydroxy-2,3-dihydro-1-benzofuran-3-carbonyl]oxy-3-(3,4-dihydroxyphenyl)propanoic	6.199217	537.1019	71836.9375	1063767.25	C27H22O12	2-arylbenzofuran flavonoids	HHKGQYGYEDKXNE-XBXARRHUSA-N	O=C(O)C=CC1=CC=C(O)C=2OC(C3=CC=C(O)C(O)=C3)C(C(=O)OC(C(=O)O)CC4=CC=C(O)C(O)=C4)C12
D-Tetrahydropalmatine	6.448717	356.1836	14163.5625	176223.8594	C21H25NO4	Protoberberine alkaloids and derivatives	AEQDJSLRWYMAQI-KRWDZBQOSA-N	O(C1=CC=C2C(=C1OC)CN3CCC4=CC(OC)=C(OC)C=C4C3C2)C
Melezitose	1.279333	527.154	1859.4375	30185.14063	C18H32O16	Oligosaccharides	QWIZNVHXZXRPDR-WSCXOGSTSA-N	OCC1OC(OC2C(O)C(OC2(OC3OC(CO)C(O)C(O)C3O)CO)CO)C(O)C(O)C1O
2-Cyclohexen-1-one, 3,5,5-trimethyl-4-[3-[(6-O-beta-D-xylopyranosyl-beta-D-glucopyranosyl)oxy]butyl]-	6.11905	549.254	28896.25	396603.4688	C24H40O11	Fatty acyl glycosides of mono- and disaccharides	LYJZPTUVRHPVNB-WRZQMYAXSA-N	O=C1C=C(C)C(CCC(OC2OC(COC3OCC(O)C(O)C3O)C(O)C(O)C2O)C)C(C)(C)C1
Vanillic acid	3.4422	167.0359	3694	92256.96094	C8H8O4	M-methoxybenzoic acids and derivatives	WKOLLVMJNQIZCI-UHFFFAOYSA-N	COC1=CC(=CC=C1O)C(O)=O
(1S,8R,9R)-8-hydroxy-4-(propan-2-ylidene)-10-oxatricyclo[7.2.1.01,5]dodecane-8-carboxylic acid	11.63342	265.1476	10044.5625	313407.9063	C15H22O4	Alpha hydroxy acids and derivatives	IOYVXXQKVQKQIG-ZJPTYJIISA-N	O=C(O)C1(O)CCC2C(=C(C)C)CCC32COC1C3
4-ACETOXYPHENOL	3.521867	151.042	5400.1875	108107.9844	C8H8O3	Phenol esters	HBMCQTHGYMTCOF-UHFFFAOYSA-N	O=C(OC1=CC=C(O)C=C1)C
Luteolin	5.565767	287.0521	4173.125	68678.04688	C15H10O6	Flavone O-glycosides	IQPNAANSBPBGFQ-UHFFFAOYSA-N	O=C1C=C(OC=2C=C(O)C=C(O)C1=2)C3=CC=C(O)C(O)=C3
dihydroferulic acid	5.396767	195.066	40206.375	495977.375	C10H12O4	Phenylpropanoic acids	BOLQJTPHPSDZHR-UHFFFAOYSA-N	O=C(O)CCC1=CC=C(O)C(OC)=C1
kaempferol 7-O-glucoside	5.717067	447.0901	4307.0625	73978.78906	C21H20O11		YPWHZCPMOQGCDQ-HMGRVEAOSA-N	O=C1C(O)=C(OC2=CC(OC3OC(CO)C(O)C(O)C3O)=CC(O)=C12)C=4C=CC(O)=CC4
Dehydrocorydaline	7.082517	366.1686	198527.25	2528167.25	C22H24NO4	Protoberberine alkaloids and derivatives	RFKQJTRWODZPHF-UHFFFAOYSA-N	COC1=C(OC)C2=C[N+]3=C(C(C)=C2C=C1)C1=CC(OC)=C(OC)C=C1CC3
[(1S,3R,5R,6S,8S)-3-{[(2S,3R,4S,5S,6R)-6-[(benzoyloxy)methyl]-3,4,5-trihydroxyoxan-2-yl]oxy}-6-hydroxy-8-methyl-9,10-dioxatetracyclo[4.3.1.0?,?.0?,?]decan-2-yl]methyl benzoate	7.438833	607.1733	16507.1875	222747.3125	C30H32O12	Terpene glycosides	LATYEZNGPQKAIK-BRVOSZROSA-N	O=C(OCC1OC(OC23CC4C5(O)OC(OC2(C)C5)C43COC(=O)C=6C=CC=CC6)C(O)C(O)C1O)C=7C=CC=CC7
Dehydrosalsolidine	6.5682	206.1152	19532.5625	230156.9219	C12H15NO2	Dihydroisoquinolines	VASUQTGZAPZKFK-UHFFFAOYSA-N	COC1=C(OC)C=C2C(C)=NCCC2=C1
Catechol	1.736133	109.0298	6830.125	121061.4844	C6H6O2	Catechols	YCIMNLLNPGFGHC-UHFFFAOYSA-N	OC1=CC=CC=C1O
(2R,3R)-2,3-bis[[(E)-3-(3,4-dihydroxyphenyl)prop-2-enoyl]oxy]butanedioic acid	4.6463	473.0724	130824.3125	3184123.5	C22H18O12	Tetracarboxylic acids and derivatives	YDDGKXBLOXEEMN-IABMMNSOSA-N	O=C(OC(C(=O)O)C(OC(=O)C=CC1=CC=C(O)C(O)=C1)C(=O)O)C=CC2=CC=C(O)C(O)=C2
3-O-Feruloylquinic acid	4.844783	367.1025	1136999.625	24257022	C17H20O9	Quinic acids and derivatives	RAGZUCNPTLULOL-KJJWLSQTSA-N	O=C(OC1CC(O)(C(=O)O)CC(O)C1O)C=CC2=CC=C(O)C(OC)=C2
2-Isopropylmalic acid	2.0531	175.0612	83897.5625	2007757.375	C7H12O5	Hydroxy fatty acids	BITYXLXUCSKTJS-ZETCQYMHSA-N	CC(C)[C@@](O)(CC(O)=O)C(O)=O
3-[6-[[(2R,3R,4R,5S,6S)-3,5-dihydroxy-6-methyl-4-[(2S,3R,4R,5R,6S)-3,4,5-trihydroxy-6-methyloxan-2-yl]oxyoxan-2-yl]oxymethyl]-3,4,5-trihydroxyoxan-2-yl]oxy-5-hydroxy-2-(4-hydroxyphenyl)-7-methoxychromen-4-one	6.67485	753.2207	16520.375	193045.4531	C34H42O19		MQMTVWHXCSRCER-OHWKTECQSA-N	O=C1C(OC2OC(COC3OC(C)C(O)C(OC4OC(C)C(O)C(O)C4O)C3O)C(O)C(O)C2O)=C(OC5=CC(OC)=CC(O)=C15)C=6C=CC(O)=CC6
3'-hydroxygenkwanin	6.647867	301.068	11398.0625	132595.9063	C16H12O6	7-O-methylated flavonoids	RRRSSAVLTCVNIQ-UHFFFAOYSA-N	O=C1C=C(OC2=CC(OC)=CC(O)=C12)C=3C=CC(O)=C(O)C3
1,7-bis(4-hydroxyphenyl)heptane-3,5-diol	6.793684	315.1578	73810.3125	817348.125	C19H24O4	Curcuminoids	GZVIQGVWSNEONZ-UHFFFAOYSA-N	OC1=CC=C(C=C1)CCC(O)CC(O)CCC2=CC=C(O)C=C2
(1r,3R,4s,5S)-4-{[(2E)-3-(3,4-Dihydroxyphenyl)-2-propenoyl]oxy}-1,3,5-trihydroxycyclohexanecarboxylic acid	2.447583	353.0877	64070.6875	3257422.25	C16H18O9	Quinic acids and derivatives	GYFFKZTYYAFCTR-UCEFVAKBSA-N	O=C(O)C1(O)CC(O)C(OC(=O)C=CC2=CC=C(O)C(O)=C2)C(O)C1
LPC 18:2	10.91563	564.3267	1277.5625	15950.88477	C26H50NO7P	Lipids	SPJFYYJXNPEZDW-UHFFFAOYNA-N	O=C(OCC(O)COP(=O)([O-])OCC[N+](C)(C)C)CCCCCCCC=CCC=CCCCCC
4,8-dimethoxy-9H-furo[2,3-b]quinolin-7-one	5.444933	246.0751	3101.75	39611.80859	C13H11NO4			Oc1ccc2c(nc3occc3c2OC)c1OC
Cryptotanshinone	10.73048	297.1463	11052	92268.26563	C19H20O3	Tanshinones, isotanshinones, and derivatives	GVKKJJOMQCNPGB-JTQLQIEISA-N	O=C1C(=O)C2=C(OCC2C)C=3C=CC4=C(C13)CCCC4(C)C
Palmatine chloride3486-67-7	6.647867	352.153	58234.625	855267.4375	C21H22ClNO4			[Cl-].O(c1ccc2cc3-c4cc(OC)c(OC)cc4CC[n+]3cc2c1OC)C

(1S,3R,4R,5R)-1,3-dihydroxy-4,5-bis[[(E)-3-(4-hydroxy-3-methoxyphenyl)prop-2-enoyl]oxy]cyclohexane-1-carboxylic acid	7.076334	543.1522	16435.25	214870.2344	C27H28O12	Quinic acids and derivatives	WCIDSNIXNCYSPH-AYCJRBPQSA-N	O=C(OC1CC(O)(C(=O)O)CC(O)C1OC(=O)C=CC2=CC=C(O)C(OC)=C2)C=CC3=CC=C(O)C(OC)=C3
3-Phenyllactic acid	5.396767	165.0561	10205.75	134514.75	C9H10O3	Phenylpropanoic acids	VOXXWSYKYCBWHO-UHFFFAOYNA-N	O=C(O)C(O)CC=1C=CC=CC1
Eleutheroside E	5.317767	787.2654	45259.9375	533023.25	C34H46O18	Lignols	FFDULTAFAQRACT-UHFFFAOYNA-N	COC1=CC(=CC(OC)=C1OC1OC(CO)C(O)C(O)C1O)C1OCC2C1COC2C1=CC(OC)=C(OC2OC(CO)C(O)C(O)C2O)C(OC)=C1
3-Hydroxy-4-methoxycinnamic acid (isoferulic acid)	5.555583	193.0527	32981.3125	477973.125	C10H10O4	Hydroxycinnamic acids	QURCVMIEKCOAJU-HWKANZROSA-N	COC1=CC=C(\C=C\C(O)=O)C=C1O
Hydroxysuberic acid	5.08095	189.0747	15322.8125	279791.375	C8H14O5	Organic acids	ARJZZFJXSNJKGR-UHFFFAOYNA-N	O=C(O)CCCCC(O)CC(=O)O
4-((3S,5R,8R,9S,10S,12R,13S,14S,17R)-3-(((2R,4S,5S,6R)-5-(((2S,4S,5S,6R)-5-(((2S,4S,5S,6R)-4,5-dihydroxy-6-methyltetrahydro-2H-pyran-2-yl)oxy)-4-hydroxy-6-methyltetrahydro-2H-pyran-2-yl)oxy)-4-hydroxy-6-methyltetrahydro-2H-pyran-2-yl)oxy)-12,14-dihydroxy-10,13-dimethylhexadecahydro-1H-cyclopenta[a]phenanthren-17-	9.01325	803.4158	2951.0625	39209.93359	C41H64O14	Cardenolide glycosides and derivatives	LTMHDMANZUZIPE-PUGKRICDSA-N	O=C1OCC(=C1)C2CCC3(O)C4CCC5CC(OC6OC(C)C(OC7OC(C)C(OC8OC(C)C(O)C(O)C8)C(O)C7)C(O)C6)CCC5(C)C4CC(O)C23C
Nordihydroguaiaretic acid	8.6531	325.1398	2088.875	44308.65625	C18H22O4	Dibenzylbutane lignans	HCZKYJDFEPMADG-UHFFFAOYSA-N	OC1=CC=C(C=C1O)CC(C)C(C)CC2=CC=C(O)C(O)=C2
Coumaroyl quinic acid (isomer of 758, 759)	3.60185	337.0916	63023.125	1745790.75	C16H18O8	Quinic acid and derivatives	XWRHBGVVCOSNKO-UHFFFAOYNA-N	O=C(OC1C(O)CC(O)(C(=O)O)CC1(O))C=CC2=CC=C(O)C=C2
piperazine-2,5-dione	7.760317	115.0528	10506.8125	209363.5469	C4H6N2O2	Alpha amino acids and derivatives	BXRNXXXXHLBUKK-UHFFFAOYSA-N	O=C1NCC(=O)NC1
(1R,3R,4S,5R)-1,3,4-trihydroxy-5-[(E)-3-(4-hydroxy-3-methoxyphenyl)prop-2-enoyl]oxycyclohexane-1-carboxylic acid	2.211433	367.1033	852470.1875	26168740	C17H20O9	Quinic acids and derivatives	RAGZUCNPTLULOL-KQJPBSFVSA-N	O=C(OC1CC(O)(C(=O)O)CC(O)C1O)C=CC2=CC=C(O)C(OC)=C2
2-Methylindole	1.596317	132.079	1600.5	41897.08594	C9H9N	Indoles	BHNHHSOHWZKFOX-UHFFFAOYSA-N	CC1=CC2=CC=CC=C2N1
Cyanidin	5.085134	287.0522	2181.6875	33605.33203	C15H11O6	7-hydroxyflavonoids	VEVZSMAEJFVWIL-UHFFFAOYSA-O	OC1=CC2=[O+]C(=C(O)C=C2C(O)=C1)C1=CC(O)=C(O)C=C1
Emodin	9.798017	269.0446	2986.375	35570.79297	C15H10O5	Hydroxyanthraquinones	RHMXXJGYXNZAPX-UHFFFAOYSA-N	CC1=CC2=C(C(O)=C1)C(=O)C1=C(C=C(O)C=C1O)C2=O
(2R,3R,4S,5S,6R)-2-[[6-hydroxy-4-(4-hydroxy-3,5-dimethoxyphenyl)-3-(hydroxymethyl)-5,7-dimethoxy-1,2,3,4-tetrahydronaphthalen-2-yl]methoxy]-6-(hydroxymethyl)oxane-3,4,5-triol	5.436417	627.2277	30723.625	394011.0938	C28H38O13	Lignan glycosides	PACBNJFGEWTGCE-OVYNBQGTSA-N	OC1=C(OC)C=C(C=C1OC)C2C3=C(OC)C(O)=C(OC)C=C3CC(COC4OC(CO)C(O)C(O)C4O)C2CO
2,5-DIHYDROXYBENZOIC ACID	2.923217	153.018	3344.0625	118962.6719	C7H6O4	Hydroxybenzoic acid derivatives	WXTMDXOMEHJXQO-UHFFFAOYSA-N	OC(=O)C1=C(O)C=CC(O)=C1
(2R,3S,4S,5R,6S)-2-[(3,4,5-trihydroxyoxan-2-yl)oxymethyl]-6-(3,4,5-trimethoxyphenoxy)oxane-3,4,5-	2.450933	501.1542	3083	70316.26563	C20H30O13	Phenolic glycosides	GGIDHIBVLYVTAU-QGPLIYBISA-N	OC1COC(OCC2OC(OC=3C=C(OC)C(OC)=C(OC)C3)C(O)C(O)C2O)C(O)C1O
5,7,8-trihydroxy-2-phenylchromen-4-one	6.608033	271.0572	2237.5	26217.12695	C15H10O5	Flavones	ZFKKRRMUPBBYRS-UHFFFAOYSA-N	O=C1C=C(OC=2C(O)=C(O)C=C(O)C12)C=3C=CC=CC3
Licoflavone A	8.81325	323.1242	9524.8125	129534.9375	C20H18O4	6-prenylated flavones	HJGURBGBPIKRER-UHFFFAOYSA-N	O=C1C=C(OC2=CC(O)=C(C=C12)CC=C(C)C)C=3C=CC(O)=CC3
Glabrol	4.333833	393.209	3296.25	47967.16406	C25H28O4	8-prenylated flavanones	CUFAXDWQDQQKFF-UHFFFAOYSA-N	O=C1C2=CC=C(O)C(=C2OC(C3=CC=C(O)C(=C3)CC=C(C)C)C1)CC=C(C)C
Gomisin O	8.28445	439.1687	1468.9375	17495.25391	C23H28O7	Hydrolyzable tannins	GWDFJIBHVSYXQL-SYTFOFBDSA-N	OC1C=2C=C(OC)C(OC)=C(OC)C2C3=C(OC)C=4OCOC4C=C3CC(C)C1C
1h-indole-3-butanoic acid	4.8868	226.0827	3815.5625	51962.16016	C12H13NO2	3-alkylindoles	JTEDVYBZBROSJT-UHFFFAOYSA-N	O=C(O)CCCC1=CNC=2C=CC=CC21
(4S,5Z,6S)-4-(2-methoxy-2-oxoethyl)-5-[2-[(E)-3-phenylprop-2-enoyl]oxyethylidene]-6-[(2S,3R,4S,5S,6R)-3,4,5-trihydroxy-6-(hydroxymethyl)oxan-2-yl]oxy-	10.7963	233.1528	3073.625	51261.53906	C15H22O2	Eremophilane, 8,9-secoeremophilane and furoeremophilane sesquiterpenoids	FMWJHXDPCQWVEJ-JVLSTEMRSA-N	O=C(O)C(=C)C1CC=C2CCCC(C)C2(C)C1
(2S,3R,4S,5S,6R)-2-[4-[[(3S,4R,5S)-3-hydroxy-5-(4-hydroxy-3-methoxyphenyl)-4-(hydroxymethyl)oxolan-3-yl]methyl]-2-methoxyphenoxy]-6-(hydroxymethyl)oxane-3,4,5-	4.097084	561.1887	1141	25836.91406	C26H34O12	Lignan glycosides	SXGSYHDLSPXCMU-CTQTXEDXSA-N	OC1=CC=C(C=C1OC)C2OCC(O)(CC3=CC=C(OC4OC(CO)C(O)C(O)C4O)C(OC)=C3)C2CO
L-5-Oxoproline	1.453967	128.0366	25446.5	384904.625	C5H7NO3	Alpha amino acids and derivatives	ODHCTXKNWHHXJC-VKHMYHEASA-N	OC(=O)[C@@H]1CCC(O)=N1
Corosolic acid	10.71713	471.3476	1991.75	24113.33984	C30H48O4	Triterpenoids	HFGSQOYIOKBQOW-UHFFFAOYNA-N	CC1CCC2(CCC3(C)C(=CCC4C5(C)CC(O)C(O)C(C)(C)C5CCC34C)C2C1C)C(O)=O
Gallic acid	1.533633	169.016	63162.125	773518.75	C7H6O5	Gallic acids	LNTHITQWFMADLM-UHFFFAOYSA-N	O=C(O)C1=CC(O)=C(O)C(O)=C1
beta-D-Glucopyranoside, 2-methoxy-4-[tetrahydro-3a,6a-dihydroxy-4-(4-hydroxy-3-methoxyphenyl)-1H,3H-furo[3,4-c]furan-1-yl]phenyl	3.1449	575.1716	2595.8125	137284.5781	C26H32O13	Lignan glycosides	IBQVOWQTGYYRJP-CPNFYLCOSA-N	OC1=CC=C(C=C1OC)C2OCC3(O)C(OCC23O)C4=CC=C(OC5OC(CO)C(O)C(O)C5O)C(OC)=C4
Caffeoyl quinic acid (isomer of 831, 833, 834)	1.736133	353.0876	639352.75	12859133	C16H18O9	Quinic acid and derivatives	CWVRJTMFETXNAD-UHFFFAOYNA-N	O=C(OC1CC(O)(C(=O)O)CC(O)C1(O))C=CC=2C=CC(O)=C(O)C=2
5,9-dihydroxy-7-(hydroxymethyl)-5,7-dimethyl-4,5a,6,8,8a,9-hexahydro-1H-azuleno[5,6-c]furan-3-one	7.237817	281.1399	10812.3125	310909.75	C15H22O5		DBKIEMOKQWYZOA-UHFFFAOYSA-N	O=C1OCC2=C1CC(O)(C)C3CC(C)(CO)CC3C2O
O-methylarmepavine	6.528217	328.1891	2566.75	32903.58203	C20H25NO3		LZJWNVLTWYMMDJ-GOSISDBHSA-N	O(C1=CC=C(C=C1)CC2C3=CC(OC)=C(OC)C=C3CCN2C)C
AZELAIC ACID	6.319033	187.0973	76040.125	818541.5	C9H16O4	Medium-chain fatty acids	BDJRBEYXGGNYIS-UHFFFAOYSA-N	O=C(O)CCCCCCCC(=O)O
3-Methyloxyindole	3.889683	148.0742	24987.3125	1336206.25	C9H9NO	Indolines	BBZCPUCZKLTAJQ-UHFFFAOYNA-N	OC1=NC=2C=CC=CC2C1C

Flavokawain A	6.050567	337.0925	9346.1875	112968.1172	C18H18O5	2'-Hydroxychalcones	CGIBCVBDFUTMPT-RMKNXTFCSA-N	O=C(C=CC1=CC=C(OC)C=C1)C=2C(O)=CC(OC)=CC2OC
Gardenin B	9.54855	359.1227	2098.875	19463.37109	C19H18O7	8-O-methylated flavonoids	LXEVSYZNYDZSOB-UHFFFAOYSA-N	O=C1C=C(OC2=C(OC)C(OC)=C(OC)C(O)=C12)C=3C=CC(OC)=CC3
Acetylleucine (isomer of 165)	5.08095	172.0975	1541.8125	19560.56836	C8H15NO3	Amino acids	WXNXCEHXYPACJF-UHFFFAOYNA-N	O=C(O)C(NC(=O)C)CC(C)C
(R)-4-aminoisoxazolidin-3-one	7.800817	103.0536	3344.625	50665.29297	C3H6N2O2	Alpha amino acids and derivatives	DYDCUQKUCUHJBH-UWTATZPHSA-N	O=C1NOCC1N
Epiberberine	9.54855	337.1388	1567.125	15889.74219	C20H18NO4+	Protoberberine alkaloids and derivatives	FPJQGFLUORYYPE-UHFFFAOYSA-N	O(C=1C=C2C=3C=C4C=CC=5OCOC5C4=C[N+]3CCC2=CC1OC)C
Hydroxysebacic acid	5.799067	217.1078	9730.4375	109765.0469	C10H18O5	Organic acids	OQYZCCKCJQWHIE-UHFFFAOYNA-N	O=C(O)CCCCCCC(O)CC(=O)O
C20H34O11	5.799067	449.1999	7647.75	132893.2656	C20H34O11	Formula predicted		
Ursolic Acid	12.97968	455.3499	5257.75	59654.35938	C30H48O3	Triterpenoids	WCGUUGGRBIKTOS-GPOJBZKASA-N	O=C(O)C12CCC(C)C(C)C2C3=CCC4C5(C)CCC(O)C(C)(C)C5CCC4(C)C3(C)CC1
(9R,10R)-10-acetoxy-8,8-dimethyl-2-oxo-2,8,9,10-tetrahydropyrano[2,3-f]chromen-9-yl 2-methylbutanoate	1.842467	389.1722	1291.125	26193.94531	C21H24O7	Angular pyranocoumarins	GVBNSPFBYXGREE-CDDDFZJFSA-N	O=C1OC2=C(C=C1)C=CC=3OC(C)(C)C(OC(=O)C(C)CC)C(OC(=O)C)C32
Higenamine	3.711367	272.1255	4528.1875	154445.875	C16H17NO3	Benzylisoquinolines	WZRCQWQRFZITDX-AWEZNQCLSA-N	OC1=CC=C(C=C1)CC2NCCC3=CC(O)=C(O)C=C32
methyl 2-((2-methyl-4-oxo-3-phenoxy-4H-chromen-7-yl)oxy)acetate	4.7283	341.0985	6470.75	100342.6797	C19H16O6	Chromones	AKNHFZVXJDNNJF-UHFFFAOYSA-N	O=C(OC)COC1=CC=C2C(OC(=C(OC=3C=CC=CC3)C2=O)C)=C1
Paeoniflorin	5.717067	525.1598	84121.0625	1486115.125	C23H28O11	Terpene glycosides	YKRGDOXKVOZESV-UHFFFAOYNA-N	CC12CC3(O)OC(O1)C1(COC(=O)C4=CC=CC=C4)C3CC21OC1OC(CO)C(O)C(O)C1O
(2R,3S,4S,5R,6S)-2-[[(2S,3R,4R)-3,4-dihydroxy-4-(hydroxymethyl)oxolan-2-yl]oxymethyl]-6-(3,4,5-trimethoxyphenoxy)oxane-3,4,5-	2.8434	523.1649	17281.6875	433647.25	C20H30O13	Phenolic glycosides	CKGKQISENBKOCA-NHBPFIHPSA-N	OCC1(O)COC(OCC2OC(OC=3C=C(OC)C(OC)=C(OC)C3)C(O)C(O)C2O)C1O
Tetrasaccharides (Hex-Hex-Hex-Hex)	1.295317	711.2183	45359.1875	571165.75	C24H42O21	Sugars	UQZIYBXSHAGNOE-UHFFFAOYNA-N	OCC4OC(OCC3OC(OCC2OC(OC1(OC(CO)C(O)C1(O))(CO))C(O)C(O)C2(O))C(O)C(O)C3(O))C(O)C(O)C4(O)
Secoisolariciresinol	6.516867	361.1646	23906.9375	299409.9063	C20H26O6	Dibenzylbutanediol lignans	PUETUDUXMCLALY-UHFFFAOYNA-N	OC1=CC=C(C=C1OC)CC(CO)C(CO)CC2=CC=C(O)C(OC)=C2
LPC 16:0	11.43427	540.3271	1127.625	12735.92871	C24H50NO7P	Lipids	ASWBNKHCZGQVJV-UHFFFAOYNA-N	O=C(OCC(O)COP(=O)([O-])OCC[N+](C)(C)C)CCCCCCCCCCCCCCC
Ellagic Acid	5.476083	300.9958	10619.3125	178773.1094	C14H6O8	Hydrolyzable tannins	AFSDNFLWKVMVRB-UHFFFAOYSA-N	O=C1OC2=C(O)C(O)=CC=3C(=O)OC=4C(O)=C(O)C=C1C4C23
pseudolaric acid B	7.800817	455.1631	1817.75	20739.26758	C23H28O8	Diterpene lactones	VDGOFNMYZYBUDT-LEGJBDPMSA-N	O=C(O)C(=CC=CC1(OC(=O)C23CC=C(C(=O)OC)CCC3(OC(=O)C)C1CC2)C)C
C21H36O11	5.920067	509.2235	23906.4375	533810.6875	C21H36O11	Formula predicted		
FA 18:2+3O	7.684467	327.2151	342336.6875	4899850	C18H32O5	Oxidized fatty acids	MKYUCBXUUSZMQB-UHFFFAOYNA-N	O=C(O)CCCCCCCC(O)C=CC(O)C(O)CC=CCC
Phenanthridine	7.00385	180.0796	1348.25	17834.09766	C13H9N	Phenanthridines and derivatives	RDOWQLZANAYVLL-UHFFFAOYSA-N	C1=CC=C2C(=C1)C=NC1=CC=CC=C21
[5-acetyloxy-1,7-bis(3,4-dihydroxyphenyl)heptan-3-yl] acetate	7.804966	431.1686	509588.9375	5757767	C23H28O8	Linear diarylheptanoids	BWSFBLYFHGZBRQ-UHFFFAOYSA-N	O=C(OC(CCC1=CC=C(O)C(O)=C1)CC(OC(=O)C)CCC2=CC=C(O)C(O)=C2)C
C12-AE1S (TENTATIVE)	12.24505	309.1689	1237.75	34238.98438	C14H30O5S	Sulfuric acid monoesters	QTDIEDOANJISNP-UHFFFAOYSA-N	CCCCCCCCCCCCOCCOS(O)(=O)=O
Flavokawain B	6.40905	307.0819	15569.1875	210730.2656	C17H16O4	2'-Hydroxychalcones	QKQLSQLKXBHUSO-CMDGGOBGSA-N	O=C(C=CC=1C=CC=CC1)C=2C(O)=CC(OC)=CC2OC
Palmitic acid (NMR)	14.4361	255.2311	1648.3125	15661.74707	C16H32O2	Long-chain fatty acids	IPCSVZSSVZVIGE-UHFFFAOYSA-N	CCCCCCCCCCCCCCCC(O)=O
4-	3.003217	121.03	2482	78891.57031	C7H6O2			O=Cc1ccc(O)cc1
Maltol	5.244617	127.0526	1790.6875	34639.42578	C6H6O3	Pyranones and derivatives	XPCTZQVDEJYUGT-UHFFFAOYSA-N	O=C1C=COC(=C1O)C
2-[(2S,4aR,8aS)-2-hydroxy-4a-methyl-8-methylidene-3,4,5,6,7,8a-hexahydro-1H-naphthalen-2-yl]prop-2-enoic acid	8.08295	249.1482	127162.9375	1595838.25	C15H22O3	Eudesmane, isoeudesmane or cycloeudesmane sesquiterpenoids	WQMBCGHXXVLQTE-CFVMTHIKSA-N	O=C(O)C(=C)C1(O)CCC2(C)CCCC(=C)C2C1
1-(3,4-Dimethoxycinnamoyl)piperidine	2.611933	298.1408	39756.75	784431.6875	C16H21NO3	Cinnamic acids and derivatives	RDNRIBOBEOTFJG-VQHVLOKHSA-N	O=C(C=CC1=CC=C(OC)C(OC)=C1)N2CCCCC2
Episyringaresinol 4'-O-beta-D-glncopyranoside	6.050567	603.203	1752.6875	20582.67773	C28H36O13	Lignan glycosides	WEKCEGQSIIQPAQ-QVYKXMJQSA-N	OC1=C(OC)C=C(C=C1OC)C2OCC3C(OCC23)C4=CC(OC)=C(OC5OC(CO)C(O)C(O)C5O)C(OC)=C4
C14H24O10	4.6463	351.1298	6752.875	92687.53906	C14H24O10	Formula predicted		
6-Methylcoumarin	8.40545	161.0577	1519.5625	23406.36133	C10H8O2	Coumarins and derivatives	FXFYOPQLGGEACP-UHFFFAOYSA-N	O=C1OC=2C=CC(=CC2C=C1)C
(3S,4R,6aR,6bS,8aS,11R,12R,14bR)-3,12-dihydroxy-4,6a,6b,11,12,14b-hexamethyl-8a-[3,4,5-trihydroxy-6-(hydroxymethyl)oxan-2-yl]oxycarbonyl-1,2,3,4a,5,6,7,8,9,10,11,12a,14,14a-tetradecahydropicene-4-carboxylic acid	8.60075	663.372	14391.6875	182800.5938	C36H56O11		AWFZJSUJFSUBQU-FZEZCDDVSA-N	O=C(O)C1(C)C(O)CCC2(C)C1CCC3(C)C2CC=C4C5C(O)(C)C(C)CCC5(C(=O)OC6OC(CO)C(O)C(O)C6O)CCC43C
FA 18:3+2O	9.1224	309.2059	37524.375	908398.4375	C18H30O4	Oxidized fatty acids	BJIKXWWZEPAKQC-UHFFFAOYNA-N	O=C(O)CCCCCCCC1OC1(C=CC(=O)CCCCC)
2-Piperidone	7.2405	100.076	4669.625	55392.38672	C5H9NO	Piperidinones	XUWHAWMETYGRKB-UHFFFAOYSA-N	C1CCN=C(C1)O
trans-5-O-Caffeoylquinic acid	1.775783	353.104	67714.8125	1424326.375	C16H18O9	Quinic acids and derivatives	CWVRJTMFETXNAD-PCNKEKKESA-N	O=C(O)C1(O)CC(O)C(O)C(OC(=O)C=CC2=CC=C(O)C(O)=C2)C1
FA 18:1+3O	8.4026	329.2336	167850	4333737.5	C18H34O5	Oxidized fatty acids	MDIUMSLCYIJBQC-UHFFFAOYNA-N	O=C(O)CCCCCCCC(O)C=CC(O)C(O)CCCCC
(1S,4aS,7S,7aS)-7-hydroxy-7-methyl-1-[(2S,3R,4S,5S,6R)-3,4,5-trihydroxy-6-(hydroxymethyl)oxan-2-yl]oxy-4a,5,6,7a-tetrahydro-1H-cyclopenta[c]pyran-4-carboxylic	1.574133	375.127	19024.25	279287.6563	C16H24O10		VLCHQFXSBHIBRV-NJPMDSMTSA-N	O=C(O)C1=COC(OC2OC(CO)C(O)C(O)C2O)C3C1CCC3(O)C
Succinic acid (not validated)	1.493633	117.0205	38085.1875	536757.1875	C4H6O4	Organic acids	KDYFGRWQOYBRFD-UHFFFAOYSA-N	O=C(O)CCC(=O)O
Homogentisic acid	1.6553	167.0359	9123.125	139138.0938	C8H8O4	2(hydroxyphenyl)acetic acids	IGMNYECMUMZDDF-UHFFFAOYSA-N	OC(=O)CC1=CC(O)=CC=C1O
5-Aminosalicylic??acid	2.963217	152.0361	2546.875	47657.24609	C7H7NO3	Aminobenzoic acids	KBOPZPXVLCULAV-UHFFFAOYSA-N	O=C(O)C1=CC(N)=CC=C1O
Schisanhenol	8.40545	425.1934	3294.875	37848.03125	C23H30O6	Hydrolyzable tannins	FYSHYFPJBONYCQ-OLZOCXBDSA-N	OC1=C(OC)C(OC)=CC2=C1C3=C(OC)C(OC)=C(OC)C=C3CC(C)C(C)C2
Anwuligan	7.082517	351.1439	192393	2409359.75	C20H24O4	Dibenzylbutane lignans	QDDILOVMGWUNGD-KGLIPLIRSA-N	OC1=CC=C(C=C1OC)CC(C)C(C)CC2=CC=C3OCOC3=C2
Malonic acid	4.688817	105.0322	3153.625	54548.19922	C3H4O4	Dicarboxylic acids and derivatives	OFOBLEOULBTSOW-UHFFFAOYSA-N	O=C(O)CC(=O)O
Indole-3-carbinol	1.596317	130.0636	1240.875	30498.46289	C9H9NO	3-alkylindoles	IVYPNXXAYMYVSP-UHFFFAOYSA-N	OCC1=CNC2=CC=CC=C12
2-Hyroxybiphenyl	7.760317	171.0772	1373.625	19532.46875	C12H10O	Biphenyls and derivatives	LLEMOWNGBBNAJR-UHFFFAOYSA-N	OC1=CC=CC=C1C1=CC=CC=C1
3-[(E)-2-(3-hydroxyphenyl)ethenyl]-5-	8.08295	241.0842	2340.875	26665.97656	C15H14O3	Stilbenes	VANIIUGEHGLNHB-AATRIKPKSA-N	OC1=CC=CC(C=CC=2C=C(O)C=C(OC)C2)=C1
2-(5-methoxy-1H-indol-3-yl)acetic acid	3.1049	206.079	1917.3125	50746.70703	C11H11NO3	Indole-3-acetic acid derivatives	COCNDHOPIHDTHK-UHFFFAOYSA-N	O=C(O)CC1=CNC=2C=CC(OC)=CC21
(1R,2R,5R,5'S,6S,8aS)-6-(acetyloxy)-2,5,8a-trimethyl-5"-oxo-octahydro-2H-dispiro[naphthalene-1,2':5',3"-bis(oxolane)]-5-ylmethyl acetate	4.9263	437.2354	5258.875	60325.25391	C24H36O7	Diterpene lactones	JZNGEGWFNPRYBH-URQJKXJZSA-N	O=C(OCC1(C)C(OC(=O)C)CCC2(C)C1CCC(C)C32OC4(COC(=O)C4)CC3)C

Bruceine A	5.647083	545.1982	1574.875	19586.61523	C26H34O11	Quassinoids	LPZSTPCYWWRQFU-HVUFZYMJSA-N	O=C(OC1C(=O)OC2CC3C(=C(O)C(=O)CC3(C)C4C(O)C(O)C5(OCC24C15)C(=O)OC)C)CC(C)C
4-hydroxy-4-(pyridin-2-yl)butan-2-one	6.170233	188.0685	20426.6875	233445.5	C9H11NO2	Pyridines and derivatives	NYKHQRHPWDLZTL-UHFFFAOYSA-N	O=C(C)CC(O)C1=NC=CC=C1
Crotonoside	6.8067	306.0734	112863	1877487.375	C10H13N5O5	Purine nucleosides	MIKUYHXYGGJMLM-UUOKFMHZSA-N	O=C1N=C(N)C=2N=CN(C2N1)C3OC(CO)C(O)C3O
Salicylic acid	6.279217	137.0236	14644.8125	192785.1875	C7H6O3	Salicylic acids	YGSDEFSMJLZEOE-UHFFFAOYSA-N	OC(=O)C1=CC=CC=C1O
2-Hydroxybenzaldehyde	4.325817	121.0314	10680.875	164916.2188	C7H6O2	Hydroxybenzaldehydes	SMQUZDBALVYZAC-UHFFFAOYSA-N	O=CC=1C=CC=CC1O
diacetylsongorine	7.478667	380.2193	1275	16606.88672	C22H31NO3	Napelline-type diterpenoid alkaloids	CBOSLVQFGANWTL-DVPYZRQCSA-N	O=C1CC2C3(CC1C(=C)C3O)C4CC5C6(C)CN(CC)C4C25C(O)CC6
Formononetin	5.76875	269.0788	5616.875	65710.14844	C16H12O4	4'-O-methylisoflavones	HKQYGTCOTHHOMP-UHFFFAOYSA-N	COC1=CC=C(C=C1)C1=COC2=C(C=CC(O)=C2)C1=O
Ganolactone B	5.244617	481.2617	2373	29658.39258	C27H38O6	Triterpenoids	FXUVJKGSDBTXTJ-XDNGZHBUSA-N	O=C1OC(C)(CC1)C2CC(=O)C3(C4=C(C(=O)CC23C)C5(C)CCC(O)C(C)(C)C5CC4O)C
Evodine	6.050567	352.1161	8318.8125	101473.5625	C18H19NO5	Furanoquinolines	LNJTUUHDKCPQAA-UHFFFAOYSA-N	OC(C(=C)C)COC=1C=CC2=C(N=C3OC=CC3=C2OC)C1OC
1-methoxyindole-3-carbaldehyde	6.448717	176.0691	8934.125	126270.0547	C10H9NO2	Indoles	NFGIENSPALNOON-UHFFFAOYSA-N	CON1C=C(C=O)C2=CC=CC=C12
LTD4	8.442266	495.2533	5661.4375	72943.25	C25H40N2O6S	Leukotrienes	YEESKJGWJFYOOK-IJHYULJSSA-N	CCCCC\C=C/C\C=C/C=C/C=C/[C@@H](SC[C@H](N)C(O)=NCC(O)=O)[C@@H](O)CCCC(O)=O
Caffeine	2.944067	195.0909	3790.125	107990.9922	C8H10N4O2	Xanthines	RYYVLZVUVIJVGH-UHFFFAOYSA-N	O=C1C2=C(N=CN2C)N(C(=O)N1C)C
Rutin	5.476083	609.1495	1750	22311.97852	C27H30O16		IKGXIBQEEMLURG-NVPNHPEKSA-N	O=C1C(OC2OC(COC3OC(C)C(O)C(O)C3O)C(O)C(O)C2O)=C(OC=4C=C(O)C=C(O)C14)C=5C=CC(O)=C(O)C5
7-hydroxy-2-methyl-3-phenyl-4H-chromen-4-one	6.170233	275.0698	5007.5625	63006.97656	C16H12O3	Isoflavones	BBCDTCKKROIGAB-UHFFFAOYSA-N	O=C1C2=CC=C(O)C=C2OC(=C1C=3C=CC=CC3)C
Dihydrochelerythrine	7.082517	350.1367	178282.375	2180528	C21H19NO4	Dihydrobenzophenanthridine alkaloids	ALZAZMCIBRHMFF-UHFFFAOYSA-N	O(C=1C=CC=2C3=CC=C4C=C5OCOC5=CC4=C3N(C)CC2C1OC)C
a-Linolenic acid (NMR)	12.85502	277.2144	1606	12274.22363	C18H30O2	Lineolic acids and derivatives	DTOSIQBPPRVQHS-PDBXOOCHSA-N	CC\C=C/C\C=C/C\C=C/CCCCCCCC(O)=O
8-prop-1-en-2-yl-8,9-dihydrofuro[2,3-h]chromen-2-one	8.6531	229.0834	1164.5	27272.26367	C14H12O3	Angular furanocoumarins	WLRXMMDATRQQNQ-UHFFFAOYSA-N	O=C1OC2=C(C=C1)C=CC=3OC(C(=C)C)CC32
(E)-10,10-dimethyl-10a-(3-(pentyloxy)styryl)-3,4,10,10a-tetrahydropyrimido[1,2-a]indol-2-	6.48855	405.245	2066.9375	24175.44922	C26H32N2O2	Indoles and derivatives	IEFRVMKLIFGNMU-JQIJEIRASA-N	OC1=NC2(C=CC=3C=CC=C(OCCCCC)C3)N(C=4C=CC=CC4C2(C)C)CC1
C21H28N4O6	4.325817	431.1896	21727.3125	294224.625	C21H28N4O6	Formula predicted		
Scoulerine6451-73-6	5.324616	328.1531	1519.875	22153.70898	C19H21NO4			Oc1cc2c(cc1OC)CCN3Cc4c(O)c(OC)ccc4CC23
(2R,3R,4S,5S,6R)-2-octoxy-6-[[(2S,3R,4S,5R)-3,4,5-trihydroxyoxan-2-yl]oxymethyl]oxane-3,4,5-triol	6.913517	469.223	3797.6875	57955.72266	C19H36O10	Fatty acyl glycosides of mono- and disaccharides	IBSNNVHJJNLMJW-BMVMOQKNSA-N	OC1COC(OCC2OC(OCCCCCCCC)C(O)C(O)C2O)C(O)C1O
HYDRANGENOSIDE C	7.56415	577.2221	2569.4375	34376.83203	C29H38O12		GGNFGJZLCXTJLH-UHFFFAOYSA-N	O=C(OC)C1=COC(OC2OC(CO)C(O)C(O)C2O)C(C=C)C1CC3OC(CC(=O)C3)CCC4=CC=C(O)C=C4
1,7-bis(3,4-dihydroxyphenyl)heptan-3-yl	7.484317	373.1628	360999	4135917.5	C21H26O6	Linear diarylheptanoids	UWNADLMLMRTPFL-UHFFFAOYSA-N	O=C(OC(CCC1=CC=C(O)C(O)=C1)CCCCC2=CC=C(O)C(O)=C2)C
hyperoside	5.59575	463.0866	3019.375	50256.49219	C21H20O12		OVSQVDMCBVZWGM-DTGCRPNFSA-N	O=C1C(OC2OC(CO)C(O)C(O)C2O)=C(OC=3C=C(O)C=C(O)C13)C=4C=CC(O)=C(O)C
INDOLE	1.759967	118.0632	3821	86490.35938	C8H7N	Indoles	SIKJAQJRHWYJAI-UHFFFAOYSA-N	N1C=CC2=CC=CC=C12
3-(4-methoxy-phenyl)-isochromen-1-one	8.81325	253.085	1401.5625	23334.40234	C16H12O3	Isocoumarins and derivatives	LCTOHXUIVJEHSV-UHFFFAOYSA-N	O=C1OC(=CC=2C=CC=CC12)C=3C=CC(OC)=CC3
4'-METHOXYFLAVONE	5.687917	253.083	2002.875	33540.86328	C16H12O3		OMICQBVLCVRFGN-UHFFFAOYSA-N	O=C1C=C(OC=2C=CC=CC12)C=3C=CC(OC)=CC3
Irisxanthone	7.076334	435.0916	3418.5	48957.10156	C20H20O11		MTQVPZUZBBTLNO-HSLVGEKZSA-N	O=C1C2=CC=C(O)C(OC)=C2OC3=CC(O)=C(C(O)=C13)C4OC(CO)C(O)C(O)C4O
(1S,4aS,7S,7aS)-7-hydroxy-7-methyl-1-[(2S,3R,4S,5S,6R)-3,4,5-trihydroxy-6-[(4-hydroxybenzoyl)oxymethyl]oxan-2-yl]oxy-4a,5,6,7a-tetrahydro-1H-cyclopenta[c]pyran-4-carboxylic acid	1.81545	495.153	15847.0625	249097.4688	C23H28O12		IUXOFSAPFXGQID-KLZCBZFCSA-N	O=C(O)C1=COC(OC2OC(COC(=O)C3=CC=C(O)C=C3)C(O)C(O)C2O)C4C1CCC4(O)C
2-(3,4-dihydroxyphenyl)ethyl (1S,4aR,7aR)-4a-hydroxy-7-methyl-5-oxo-1-[(2S,3R,4S,5S,6R)-3,4,5-trihydroxy-6-(hydroxymethyl)oxan-2-yl]oxy-1,6,7,7a-tetrahydrocyclopenta[c]pyran-4-	2.171933	525.157	4764.1875	172338.3438	C24H30O13		KQBFNIVRWYGXRN-QJMXJWGSSA-N	O=C(OCCC1=CC=C(O)C(O)=C1)C2=COC(OC3OC(CO)C(O)C(O)C3O)C4C(C)CC(=O)C24O
heliocurassavicine n-oxyde	7.478667	302.2081	2538.25	52408.82813	C15H27NO5	Pyrrolizidines	DLNWZIVYKQXLTN-YOPIWALVSA-N	O=C(OCC1CC[N+]2([O-])CCCC12)C(O)(C(O)C)C(C)C
Trehalose	1.374633	341.1087	42707.4375	699833.9375	C12H22O11		HDTRYLNUVZCQOY-LIZSDCNHSA-N	OCC1OC(OC2OC(CO)C(O)C(O)C2O)C(O)C(O)C1O
Phenylacetylaspartic acid	4.325817	250.0717	3791.0625	78573.23438	C12H13NO5	Amino acids	SVFKZPQPMMZHLZ-UHFFFAOYNA-N	O=C(O)CC(NC(=O)CC1=CC=CC=C1)C(=O)O
lysergol	11.68427	277.1197	1222.5625	9110.029297	C16H18N2O	Clavines and derivatives	BIXJFIJYBLJTMK-BONVTDFDSA-N	OCC1C=C2C=3C=CC=C4NC=C(C43)CC2N(C)C1
methyl 3-[3,4-dihydroxy-5-(3-methylbut-2-enyl)phenyl]-2-[[4-hydroxy-3-(3-methylbut-2-enyl)phenyl]methyl]-4-methoxy-5-oxofuran-2-carboxylate	5.555583	521.2195	12674.125	191349.375	C30H34O8		LARSXHUKWMVHRB-UHFFFAOYSA-N	O=C1OC(C(=O)OC)(C(C=2C=C(O)C(O)=C(C2)CC=C(C)C)=C1OC)CC3=CC=C(O)C(=C3)CC=C(C)C
Tetrahydrocolumbamine	5.891417	342.1663	18059.3125	254155.4375	C20H23NO4	Protoberberine alkaloids and derivatives	KDFKJOFJHSVROC-INIZCTEOSA-N	OC=1C=C2C(=CC1OC)CCN3CC4=C(OC)C(OC)=CC=C4CC23
Isocorydine	5.4851	342.168	14668.125	200330.4063	C20H23NO4	Aporphines	QELDJEKNFOQJOY-ZDUSSCGKSA-N	OC=1C(OC)=CC=C2C1C=3C(OC)=C(OC)C=C4C3C(N(C)CC4)C2
albiflorin	5.199266	479.1535	36272.25	578461.5625	C23H28O11	Terpene glycosides	QQUHMASGPODSIW-ICECTASOSA-N	O=C(OCC12C(=O)OC3(C)CC(O)C2CC31OC4OC(CO)C(O)C(O)C4O)C=5C=CC=CC5
methyl (1S,5S,9S,10R,15R)-15-(acetyloxy)-8-hydroxy-4,5,7,10,14,14-hexamethyl-6,18-dioxo-19-oxapentacyclo[10.5.2.0?,??.0?,??.0?,?]nonadeca-3,7-diene-9-carboxylate	8.922234	499.2336	4966.625	57856.53906	C28H36O8	Steroid esters	JYWRCSAWBUAIFO-KRRNMHNDSA-N	O=C(OC1CCC23C(=O)OC(CC4(C)C2C=C(C)C5(C(=O)C(=C(O)C54C(=O)OC)C)C)C3C1(C)C)C
2-Acetylacteoside	1.279333	689.2062	3114.9375	42368.80469	C31H38O16	Coumaric acids and derivatives	ALERZNQPBWWLMW-YVMONPNESA-N	O=C(OC1C(OC(OCCC2=CC=C(O)C(O)=C2)C(OC(=O)C)C1OC3OC(C)C(O)C(O)C3O)CO)C=CC4=CC=C(O)C(O)=C4
1-(9-hydroxy-2-isopropyl-10-(2,3,4-trimethoxyphenyl)pyrrolo[3',4':6,7]azepino[4,3,2-cd]indol-8(2H,7H,10H)-yl)ethanone	8.0023	498.1866	1577.375	18160.65039	C27H29N3O5	Benzazepines	QAPJGIXRNRUPRW-UHFFFAOYSA-N	O=C(N1C(O)=C2C(=NC3=CC=CC4=C3C(=CN4C(C)C)C2C5=CC=C(OC)C(OC)=C5OC)C1)C
Asarone	10.77032	209.0947	2193.4375	19069.93555	C12H16O3	Anisoles	RKFAZBXYICVSKP-AATRIKPKSA-N	O(C=1C=C(OC)C(C=CC)=CC1OC)C
N-acetylphenylalanine	5.002117	206.0834	5390.75	83644.69531	C11H13NO3		CBQJSKKFNMDLON-UHFFFAOYSA-N	O=C(O)C(N=C(O)C)CC=1C=CC=CC1
Psoralen	8.612933	187.0378	3518.0625	66152.80469	C11H6O3	Psoralens	ZCCUUQDIBDJBTK-UHFFFAOYSA-N	O=C1OC2=CC=3OC=CC3C=C2C=C1

Limonin	8.4026	469.1861	14971.375	181095.5156	C26H30O8		KBDSLGBFQAGHBE-MSGMIQHVSA-N	O=C1OCC23C(OC(C)(C)C3CC(=O)C4(C)C2CCC5(C)C(OC(=O)C6OC645)C7=COC=C7)C1
Tenuifoliside A	1.279333	705.1788	58874.25	817537.125	C31H38O17	Coumaric acids and derivatives	VOZDDCMEFRAJHD-XMPZFKQGSA-N	O=C(OCC1OC(OC2(OC(CO)C(O)C2OC(=O)C3=CC=C(O)C=C3)CO)C(O)C(O)C1O)C=CC4=CC(OC)=C(OC)C(OC)=C4
7-methoxy-2-methyl-3-phenyl-4H-chromen-4-one	6.448717	267.0997	2908.625	36438.59375	C17H14O3	7-O-methylisoflavones	XRGWZIGGCNSFRY-UHFFFAOYSA-N	O=C1C2=CC=C(OC)C=C2OC(=C1C=3C=CC=CC3)C
L-Threonic acid hemicalcium salt	1.4143	135.0315	7416.4375	127943.3125	C4H8O5	Sugar acids and derivatives	JPIJQSOTBSSVTP-STHAYSLISA-N	OC[C@H](O)[C@@H](O)C(O)=O
C16H30O4	9.0424	285.205	2818.375	50863.24219	C16H30O4	Formula predicted		
arborinine	5.971233	308.0893	15264.5	224128.6719	C16H15NO4	Acridones	ATBZZQPALSPNMF-UHFFFAOYSA-N	O=C1C=2C=CC=CC2N(C3=CC(OC)=C(OC)C(O)=C13)C
Theophylline	9.458217	181.0623	1205.125	20679.0625	C7H8N4O2	Xanthines	ZFXYFBGIUFBOJW-UHFFFAOYSA-N	O=C1C=2NC=NC2N(C(=O)N1C)C
FA 9:0+1O	6.477366	173.1175	4354.0625	54768.73438	C9H18O3	Oxidized fatty acids	XBUXARJOYUQNTC-UHFFFAOYNA-N	O=C(O)CC(O)CCCCCC
(2S,3S,4S,5R,6R)-6-[[(3S,6aR,6bS,8aS,14bR)-4,4,6a,6b,11,11,14b-heptamethyl-8a-[(2S,3R,4S,5S,6R)-3,4,5-trihydroxy-6-(hydroxymethyl)oxan-2-yl]oxycarbonyl-1,2,3,4a,5,6,7,8,9,10,12,12a,14,14a-tetradecahydropicen-3-yl]oxy]-3,5-dihydroxy-4-[(2S,3R,4S,5R,6R)-3,4,5-	7.804966	955.4935	1487.3125	22504.75195	C48H76O19		QZMAEZWZCGBZFK-VPQYALDZSA-N	O=C(O)C1OC(OC2CCC3(C)C4CC=C5C6CC(C)(C)CCC6(C(=O)OC7OC(CO)C(O)C(O)C7O)CCC5(C)C4(C)CCC3C2(C)C)C(O)C(OC8OC(CO)C(O)C(O)C8O)C1O
4-(3,4-dihydroxyphenyl)-6,7-dihydroxynaphthalene-2-carboxylic acid	7.197	311.0533	10787.375	151091.5938	C17H12O6	Naphthalenecarboxylic acids	ZSKDVJYWOHBGNI-UHFFFAOYSA-N	O=C(O)C1=CC=2C=C(O)C(O)=CC2C(=C1)C=3C=CC(O)=C(O)C3
Tryptophanol (not validated)	4.8868	144.08	2831.1875	39591.66016	C10H11NO	Indole and derivatives	MBBOMCVGYCRMEA-UHFFFAOYSA-N	OCCC2=CNC1=CC=CC=C12
(6S,6aS,13S,13aR)-N-(benzo[d][1,3]dioxol-5-ylmethyl)-2,3,4,6,6a,7,8,9,10,12,13,13a-dodecahydro-1H-6,13-methanodipyrido[1,2-a:3',2'-e]azocine-1-carboxamide	7.962133	410.2304	1608.1875	24052.72266	C24H31N3O3	Aloperine and related alkaloids	CMDLPOPJXJYAMB-UHFFFAOYSA-N	O=C(NCC1=CC=C2OCOC2=C1)N3CCCC4=CC5CC(CN6CCCCC65)C43
2-[(3S)-3-hydroxy-4a,8-dimethyl-2,3,4,5,6,7-hexahydro-1H-naphthalen-2-yl]prop-2-enoic acid	7.7648	249.1483	503761.625	5829712.5	C15H22O3	Eudesmane, isoeudesmane or cycloeudesmane sesquiterpenoids	MMLPRYWSGSVRRN-QRJNDHJOSA-N	O=C(O)C(=C)C1CC2=C(C)CCCC2(C)CC1O
Picroside I	2.777583	515.1689	2314.875	56761.85938	C24H28O11	O-glycosyl compounds	BSYHSWKTXMTFNF-NTLZGZQOSA-N	O=C(OC1C2OC2(CO)C3C(OC=CC13)OC4OC(CO)C(O)C(O)C4O)C=CC=5C=CC=CC5
(S)-4-((7-acetamido-1,2,3-trimethoxy-9-oxo-5,6,7,9-tetrahydrobenzo[a]heptalen-10-yl)amino)-N-(3-(methylthio)phenyl)butanamide	4.9658	592.2339	20791.9375	252682.1719	C32H37N3O6S	Gamma amino acids and derivatives	HRHHAUYCXBULEA-VWLOTQADSA-N	O=C1C=C2C(=CC=C1NCCCC(=O)NC=3C=CC=C(SC)C3)C4=C(OC)C(OC)=C(OC)C=C4CCC2NC(=O)C
Gigantol	4.7283	297.1104	12580.625	207553.2969	C16H18O4	Stilbenes	SDXKZPQOVUDXIY-UHFFFAOYSA-N	OC=1C=C(OC)C=C(C1)CCC2=CC=C(OC)C(O)=C2
Yangonin	5.72825	281.0801	6619.125	88131.9375	C15H14O4	Kavalactones	XLHIYUYCSMZCCC-VMPITWQZSA-N	O=C1OC(C=CC2=CC=C(OC)C=C2)=CC(OC)=C1
methyl 1-methyl-9H-beta-carbolin-7-yl ether	9.974	213.0883	1439.1875	15256.81934	C13H12N2O	Harmala alkaloids	BXNJHAXVSOCGBA-UHFFFAOYSA-N	N=1C=CC=2C=3C=CC(OC)=CC3NC2C1C
2,4,6-Trihydroxyacetophenone	4.6863	167.0359	1887.9375	24595.60156	C8H8O4		XLEYFDVVXLMULC-UHFFFAOYSA-N	O=C(C=1C(O)=CC(O)=CC1O)C
Baicalin	5.476083	445.0764	7524.375	144479.8906	C21H18O11	Flavonoid-7-O-glucuronides	IKIIZLYTISPENI-ZFORQUDYSA-N	O[C@@H]1[C@@H](O)[C@H](OC2=C(O)C(O)=C3C(=O)C=C(OC3=C2)C2=CC=CC=C2)O[C@@H]([C@H]1O)C(O)=O
bonactin	7.719817	423.2307	1353.625	25147.67188	C21H36O7		JZCRGJSEBZCNAR-UHFFFAOYSA-N	O=C(O)C(C)C1OC(CC1)CC(OC(=O)C(C)C2OC(CC2)CC(O)CC)C
Homovanillic acid	3.003217	181.05	120562.3125	2447730.25	C9H10O4	Methoxyphenols	QRMZSPFSDQBLIX-UHFFFAOYSA-N	O=C(O)CC1=CC=C(O)C(OC)=C1
Soyasaponin Ba	7.0355	957.4686	46170.125	598833.6875	C48H78O19	Triterpene saponins	WFRQIKSNAYYUJZ-UHFFFAOYNA-N	CC1(C)CC(O)C2(C)CCC3(C)C(=CCC4C5(C)CCC(OC6OC(C(O)C(O)C6OC6OC(CO)C(O)C(O)C6OC6OC(CO)C(O)C(O)C6O)C(O)=O)C(C)(CO)C5CCC34C)C2C1
Piperine	4.530983	286.1412	27469.0625	431552.9063	C17H19NO3	Alkaloids and derivatives	MXXWOMGUGJBKIW-YPCIICBESA-N	O=C(C=CC=CC1=CC=C2OCOC2=C1)N3CCCCC3
2,3,9,10-tetramethoxy-6,8,13,13a-tetrahydro-5H-isoquinolino[2,1-b]isoquinoline	6.010917	356.2022	6928.375	113877.3984	C21H25NO4	Protoberberine alkaloids and derivatives	AEQDJSLRWYMAQI-UHFFFAOYSA-N	O(C1=CC=C2C(=C1OC)CN3CCC4=CC(OC)=C(OC)C=C4C3C2)C
2-(1,3-benzodioxol-5-yl)-1-methylquinolin-4-one	5.931083	280.0936	10579.25	137836.9531	C17H13NO3			O=c1cc(c2ccc3OCOc3c2)n(c4ccccc14)C
N6-benzyl-7H-purine-2,6-	8.81325	241.1202	1140.875	17348.84961	C12H12N6	6-alkylaminopurines	XSSREOLCRTWEPI-UHFFFAOYSA-N	N1=CNC2=C1N=C(N=C2NCC=3C=CC=CC3)N
FA 18:1+2O	9.75835	313.2359	18327.3125	375060.1875	C18H34O4	Oxidized fatty acids	CQSLTKIXAJTQGA-UHFFFAOYNA-N	O=C(O)CCCCCCCC=CCC(O)C(O)CCCCC
Columbamine	6.40905	339.143	4044.5625	124967.5859	C20H20NO4+	Protoberberine alkaloids and derivatives	YYFOFDHQVIODOQ-UHFFFAOYSA-O	OC=1C=C2C=3C=C4C=CC(OC)=C(OC)C4=C[N+]3CCC2=CC1OC
Angustine	2.247783	314.1385	5902.3125	108078.1719	C20H15N3O	Beta carbolines	FACXQEOSOVJIPD-UHFFFAOYSA-N	O=C1C=2C=NC=C(C=C)C2C=C3C=4NC=5C=CC=CC5C4CCN13
5-hydroxy-2-(4-methoxyphenyl)-3-[(2S,3R,4S,5S,6R)-3,4,5-trihydroxy-6-(hydroxymethyl)oxan-2-yl]oxy-7-[(2S,3R,4R,5R,6S)-3,4,5-trihydroxy-6-methyloxan-2-	6.793684	607.1634	4200.125	51125.12109	C28H32O15		OFKKUHQXUNAUKP-CQNMNSRFSA-N	O=C1C(OC2OC(CO)C(O)C(O)C2O)=C(OC3=CC(OC4OC(C)C(O)C(O)C4O)=CC(O)=C13)C=5C=CC(OC)=CC5
Estrone	9.5612	269.1536	4470.875	58574.75391	C18H22O2	Estrogens and derivatives	DNXHEGUUPJUMQT-UHFFFAOYNA-N	O=C1CCC2C3CCC4=CC(O)=CC=C4C3CCC12C
(2E)-3-(4-hydroxy-3-methoxyphenyl)-2-propenoic acid	9.418217	195.0787	1988	34621.86719	C10H10O4	Hydroxycinnamic acids	KSEBMYQBYZTDHS-HWKANZROSA-N	O=C(O)C=CC1=CC=C(O)C(OC)=C1
5-Fluorouracil	1.533633	129.0208	2218.0625	43019.10938	C4H3FN2O2	Halopyrimidines	GHASVSINZRGABV-UHFFFAOYSA-N	OC1=NC=C(F)C(O)=N1
(2S,3S,4S,5R,6S)-6-[2-(3,4-dihydroxyphenyl)-5-hydroxy-4-oxochromen-7-yl]oxy-3,4,5-trihydroxyoxane-2-carboxylic	5.59575	461.0679	7083.5625	152884.75	C21H18O12		VSUOKLTVXQRUSG-ZFORQUDYSA-N	O=C(O)C1OC(OC2=CC(O)=C3C(=O)C=C(OC3=C2)C=4C=CC(O)=C(O)C4)C(O)C(O)C1O
6-methoxy-4-methyl-2H-chromen-2-one	5.204783	191.0833	3730.625	54538.91016	C11H10O3	Coumarins and derivatives	KNGPIBWCWOQBEK-UHFFFAOYSA-N	O=C1OC=2C=CC(OC)=CC2C(=C1)C
D-(-)-quinic acid	4.766117	191.0563	23095.875	450722.8125	C7H12O6	Quinic acids and derivatives	AAWZDTNXLSGCEK-LNVDRNJUSA-N	O=C(O)C1(O)CC(O)C(O)C(O)C1
Diphenylcyclopropenone	4.688817	207.0788	8003.5	129952.2422	C15H10O	Benzene and substituted derivatives	HCIBTBXNLVOFER-UHFFFAOYSA-N	O=C1C(C=2C=CC=CC2)=C1C=3C=CC=CC3
2-Hydroxy-3,4-Dimethoxybenzoic Acid	6.039233	197.0454	4648.0625	66906.875	C9H10O5		CJFQIVAOBBTJCI-UHFFFAOYSA-N	O=C(O)C1=CC=C(OC)C(OC)=C1O

(6,6-Dimethylbicyclo[3.1.1]hept-2-yl)methyl 6-O-[(2R,3R,4R)-3,4-dihydroxy-4-(hydroxymethyl)tetrahydro-2-furanyl]-beta-D-glucopyranoside	6.913517	493.2227	14507.5625	319294.0938	C21H36O10	Terpene glycosides	RSKTWDAINBIMPD-MCVCZSGFSA-N	OCC1(O)COC(OCC2OC(OCC3CCC4CC3C4(C)C)C(O)C(O)C2O)C1O
Echinatin	6.170233	293.0803	1535.6875	30547.11719	C16H14O4	Retrochalcones	QJKMIJNRNRLQSS-WEVVVXLNSA-N	O=C(C=CC1=CC=C(O)C=C1OC)C2=CC=C(O)C=C2
6,7-dimethoxy-quinazoline-2,4-	9.3334	223.0741	5152.75	53152.84375	C10H10N2O4	Quinazolines	KWNQIIMVPSMYEM-UHFFFAOYSA-N	OC=1N=C(O)C=2C=C(OC)C(OC)=CC2N1
Corydaline	6.5682	370.1977	29610.9375	446725.625	C22H27NO4	Protoberberine alkaloids and derivatives	VRSRXLJTYQVOHC-QUXALOBESA-N	O(C1=CC=C2C(=C1OC)CN3CCC4=CC(OC)=C(OC)C=C4C3C2C)C
(E)-3-[4-methoxy-2-[(2S,3R,4S,5S,6R)-3,4,5-trihydroxy-6-(hydroxymethyl)oxan-2-	3.243033	401.1097	8495.4375	318322.9063	C16H20O9	Phenolic glycosides	FQWZGEBZILOCET-BJGSYIFTSA-N	O=C(O)C=CC1=CC=C(OC)C=C1OC2OC(CO)C(O)C(O)C2O
3-(2-((3-(2-carboxyethyl)-4-methyl-5-((Z)-(3-methyl-5-oxo-4-vinyl-1H-pyrrol-2(5H)-ylidene)methyl)-1H-pyrrol-2-yl)methyl)-4-methyl-5-((Z)-(4-methyl-5-oxo-3-vinyl-1H-pyrrol-2(5H)-ylidene)methyl)-1H-pyrr	5.565767	607.2321	8440.8125	92960.20313	C33H36N4O6	Bilirubins	BPYKTIZUTYGOLE-IFADSCNNSA-N	O=C(O)CCC1=C(NC(C=C2NC(=O)C(=C2C=C)C)=C1C)CC=3NC(C=C4NC(=O)C(C=C)=C4C)=C(C3CCC(=O)O)C
Rinderine	7.2405	300.1942	45976.6875	515179.5938	C15H25NO5	Alkaloids and derivatives	SFVVQRJOGUKCEG-ZRQNBYAXSA-N	CC(C)[C@](O)([C@@H](C)O)C(=O)OCC1=CCN2CC[C@H](O)[C@@H]12
Inermin	2.566083	283.0419	2523.5625	46806.17969	C16H12O5		HUKSJTUUSUGIDC-ZBEGNZNMSA-N	OC1=CC=C2C(OCC3C4=CC=5OCOC5C=C4OC23)=C1
5,6-Dimethylbenzimidazole	6.477366	145.0855	2047.6875	24995.5332	C9H10N2	Benzimidazoles	LJUQGASMPRMWIW-UHFFFAOYSA-N	N1=CNC=2C=C(C(=CC12)C)C
synephrine	1.759967	190.0838	2785.5	59164.72656	C9H13NO2	1-hydroxy-2-unsubstituted benzenoids	YRCWQPVGYLYSOX-UHFFFAOYSA-N	OC1=CC=C(C=C1)C(O)CNC
beta-D-Glucopyranoside, 2-phenylethyl 6-O-beta-D-xylopyranosyl-	5.357267	461.1616	7886	166302.1406	C19H28O10	O-glycosyl compounds	ZRGXCWYRIBRSQA-UHFFFAOYSA-N	OC1COC(OCC2OC(OCCC=3C=CC=CC3)C(O)C(O)C2O)C(O)C1O
2,3-dihydro-1H-carbazol-4(9H)-one	6.448717	208.0879	1660.1875	24927.49609	C12H11NO	Carbazoles	DSXKDTZEIWTHRO-UHFFFAOYSA-N	O=C1C=2C=3C=CC=CC3NC2CCC1
Bisphenol A	9.974	229.1194	1392.6875	14416.13477	C15H16O2	Bisphenols	IISBACLAFKSPIT-UHFFFAOYSA-N	CC(C)(C1=CC=C(O)C=C1)C1=CC=C(O)C=C1
Phillyrin	6.833517	579.2118	3347.6875	43860.75	C27H34O11	Lignan glycosides	KFFCKOBAHMGTMW-UHFFFAOYNA-N	COC1=C(OC)C=C(C=C1)C1OCC2C1COC2C1=CC(OC)=C(OC2OC(CO)C(O)C(O)C2O)C=C1
colchicine	5.891417	400.1742	2544.4375	30908.44922	C22H25NO6	Tropones	IAKHMKGGTNLKSZ-INIZCTEOSA-N	O=C1C=C2C(=CC=C1OC)C3=C(OC)C(OC)=C(OC)C=C3CCC2NC(=O)C
Kirenol	7.00385	361.2339	1213.6875	15869.95898	C20H34O4	Diterpenoids	NRYNTARIOIRWAB-JPDRSCFKSA-N	OCC(O)C1(C=C2CCC3C(C)(CO)CC(O)CC3(C)C2CC1)C
L-Tryptophan	9.889116	205.0988	8664.25	73334.28906	C11H12N2O2	Indolyl carboxylic acids and derivatives	QIVBCDIJIAJPQS-VIFPVBQESA-N	N[C@@H](CC1=CNC2=CC=CC=C12)C(O)=O
5,7-dihydroxy-6-methoxy-2-phenylchromen-4-one	8.720917	283.0609	1064.25	13096.06836	C16H12O5		LKOJGSWUMISDOF-UHFFFAOYSA-N	O=C1C=C(OC2=CC(O)=C(OC)C(O)=C12)C=3C=CC=CC3
(7,8-dimethoxy-2,2,3-trimethyl-2,3-dihydro-1H-benzo[d]azepin-4-yl)(3,4-	7.201	412.2118	4428.1875	53322.47266	C24H29NO5	Benzazepines	IINUIABWYMTUJM-UHFFFAOYSA-N	O=C(C1=CC=C(OC)C(OC)=C1)C2=CC=3C=C(OC)C(OC)=CC3CC(N2C)(C)C
[(2R,3R,4R,5R,6R)-2-[[(2R,3R,4R)-3,4-dihydroxy-4-(hydroxymethyl)oxolan-2-yl]oxymethyl]-4-[(2S,3R,4R,5R,6S)-4,5-dihydroxy-6-methyl-3-[(2S,3R,4S,5S)-3,4,5-trihydroxyoxan-2-yl]oxyoxan-2-yl]oxy-6-[2-(3,4-dihydroxyphenyl)ethoxy]-5-hydroxyoxan-3-yl] (E)-3-(3,4-	6.159217	887.295	2338.875	40382.79688	C39H52O23		NPVUMVVTBDKDDD-NTQQJVIQSA-N	O=C(OC1C(OC(OCCC2=CC=C(O)C(O)=C2)C(O)C1OC3OC(C)C(O)C(O)C3OC4OCC(O)C(O)C4O)COC5OCC(O)(CO)C5O)C=CC6=CC=C(O)C(O)=C6
1,7,8-trihydroxy-3-methyl-2,3,4,7-tetrahydro-1H-benzo[a]anthracen-12-one	5.72825	311.1256	2390	43967.85547	C19H18O4	Phenanthrols	FDVOVSCQDWKKHH-UHFFFAOYSA-N	O=C1C=2C=CC=C(O)C2C(O)C3=CC=C4C(=C13)C(O)CC(C)C4
alpha-D-Glucopyranoside, alpha-D-glucopyranosyl, 2-(2-methylbutanoate)	4.164333	471.1679	3816.875	98923.57031	C17H30O12	Acyltrehaloses	FNVXTGOURNXUCQ-NVGKCVISSA-N	O=C(OC1C(O)C(O)C(OC1OC2OC(CO)C(O)C(O)C2O)CO)C(C)CC
(S)-3-(4-hydroxyphenyl)chroman-7-ol	4.7283	265.0823	25349.4375	372799.0625	C15H14O3	Isoflavanols	ADFCQWZHKCXPAJ-GFCCVEGCSA-N	OC1=CC=C(C=C1)C2COC3=CC(O)=CC=C3C2
Tetrahydrocoptisine	5.931083	324.1219	4446.625	95649.74219	C19H17NO4	Protoberberine alkaloids and derivatives	UXYJCYXWJGAKQY-OAHLLOKOSA-N	O1C2=CC=C3C(=C2OC1)CN4CCC5=CC=6OCOC6C=C5C4C3
n-	6.369383	382.1628	1586.3125	30266.51563	C20H25NO5	Dimethoxybenzenes	KDIKNBJFJOYFNY-UHFFFAOYSA-N	O=C(NCCC1=CC=C(OC)C(OC)=C1)CC2=CC=C(OC)C(OC)=C2
beta-D-Glucopyranoside, (1R,2S,4R,5R)-5-hydroxy-1,7,7-trimethylbicyclo[2.2.1]hept-2-yl 6-O-[(2R,3R,4R)-tetrahydro-3,4-dihydroxy-4-(hydroxymethyl)-2-furanyl]-	5.971233	487.2094	3390.9375	72384.36719	C21H36O11	Terpene glycosides	BVEVSYQMQABMQM-UHFFFAOYSA-N	OCC1(O)COC(OCC2OC(OC3CC4C(O)CC3(C)C4(C)C)C(O)C(O)C2O)C1O
Octopamine, N-p-coumaroyl-	6.40905	322.1048	46497.25	657156.625	C17H17NO4	Styrenes	VATOSFCFMOPAHX-XCVCLJGOSA-N	O=C(C=CC1=CC=C(O)C=C1)NCC(O)C2=CC=C(O)C=C2
3-hydroxybenzo(a)pyrene	4.36615	267.0918	7417	165930.4844	C20H12O	Benzopyrenes	SPUUWWRWIAEPDB-UHFFFAOYSA-N	C1=CC=C2C(=C1)C=C3C=CC4=C(C=CC=5C=CC2=C3C54)O
10-Hydroxydecanoic acid	9.0424	187.1321	1857.75	21858.89453	C10H20O3	Medium-chain hydroxy acids and derivatives	YJCJVMMDTBEITC-UHFFFAOYSA-N	O=C(O)CCCCCCCCCO
Quercetin	8.481916	301.0326	1874.8125	23981.49023	C15H10O7	Flavonols	REFJWTPEDVJJIY-UHFFFAOYSA-N	OC1=CC(O)=C2C(OC(=C(O)C2=O)C2=CC(O)=C(O)C=C2)=C1
Terbutylazine-desethyl	1.636983	202.0859	1004.1875	25249.67578	C7H12ClN5	Aminotriazines	LMKQNTMFZLAJDV-UHFFFAOYSA-N	CC(C)(C)NC1=NC(Cl)=NC(=N)N1
8-[(2S,3R,4R,5S,6R)-3,4-dihydroxy-6-(hydroxymethyl)-5-[(2S,3R,4S,5S,6R)-3,4,5-trihydroxy-6-(hydroxymethyl)oxan-2-yl]oxyoxan-2-yl]-5,7-dihydroxy-	5.199266	593.1516	3050.875	45543.57813	C27H30O15		NDSUKTASTPEKBX-LXXMDOISSA-N	O=C1C=C(OC=2C1=C(O)C=C(O)C2C3OC(CO)C(OC4OC(CO)C(O)C(O)C4O)C(O)C3O)C=5C=CC(O)=CC5
Orcinol	1.81545	123.047	4969.3125	78272.0625	C7H8O2	Resorcinols	OIPPWFOQEKKFEE-UHFFFAOYSA-N	OC=1C=C(O)C=C(C1)C
[7-(3,4-dihydroxyphenyl)-1-(4-hydroxyphenyl)heptan-3-yl]	7.963783	357.1715	28551.375	328994.3125	C21H26O5	Linear diarylheptanoids	HLPXMGYELYMAQM-UHFFFAOYSA-N	O=C(OC(CCC1=CC=C(O)C=C1)CCCCC2=CC=C(O)C(O)=C2)C
Ketoprofen	3.592533	255.0992	7142.375	276111.5938	C16H14O3	Benzophenones	DKYWVDODHFEZIM-UHFFFAOYNA-N	CC(C(O)=O)C1=CC(=CC=C1)C(=O)C1=CC=CC=C1

(1S,3R,6R,7S,8S,9R,10S,13S,16S,17R)-8-tert-butyl-6,9,12,17-tetrahydroxy-16-methyl-2,4,14,19-tetraoxahexacyclo[8.7.2.0?,??.0?,?.0?.??.0??.??]nonadecane-	7.201	458.1564	1914.75	21812.46484	C20H24O11		AMOGMTLMADGEOQ-GJCYDYJKSA-N	O=C1OC2OC34C(=O)OC5C(O)C(C(C)(C)C)C2(C1O)C54C(O)C6OC(=O)C(C)C63O
3,5-Dimethoxycinnamic acid	7.279167	207.0659	10084.25	112615.8203	C11H12O4	Coumaric acids and derivatives	VLSRUFWCGBMYDJ-UHFFFAOYSA-N	COC1=CC(C=CC(O)=O)=CC(OC)=C1
2(1H)-Quinolinone, 3,4-dihydro-3,4-dihydroxy-4-(4-methoxyphenyl)-	10.73048	268.106	7847.375	66427.41406	C16H15NO4	Phenylquinolines	VJLVPUFVTPJHDI-UHFFFAOYSA-N	OC1=NC=2C=CC=CC2C(O)(C3=CC=C(OC)C=C3)C1O
DIHYDROTANSHINONE I	6.448717	279.0977	26012.0625	304568.6875	C18H14O3	Tanshinones, isotanshinones, and derivatives	HARGZZNYNSYSGJ-UHFFFAOYSA-N	O=C1C(=O)C2=C(OCC2C)C=3C=CC4=C(C=CC=C4C)C13
3,9-dihydroxy-6-oxo-7-(2-oxoheptyl)-1-pentylbenzo[b][1,4]benzodioxepine-2-carboxylic acid	8.4026	515.1895	323232.875	3753279.75	C26H30O8	Depsides and depsidones	KVTYWHGIZSCFLG-UHFFFAOYSA-N	O=C(O)C1=C(O)C=C2OC(=O)C=3C(OC2=C1CCCCC)=CC(O)=CC3CC(=O)CCCCC
Asiatic Acid	9.5612	487.3421	1859.5	53729.27734	C30H48O5	Triterpenoids	JXSVIVRDWWRQRT-UHFFFAOYSA-N	O=C(O)C12CCC(C)C(C)C2C3=CCC4C5(C)CC(O)C(O)C(C)(CO)C5CCC4(C)C3(C)CC1
2,8-Dimethyl-5,7-dimethoxychromone	9.293567	235.1096	2252.8125	24372.10156	C13H14O4		MJHNYZONVLZSNB-UHFFFAOYSA-N	O=C1C=C(OC=2C1=C(OC)C=C(OC)C2C)C
4-amino-1-((2R,3R,4S,5R)-3,4-dihydroxy-5-(hydroxymethyl)tetrahydrofuran-2-yl)pyrimidin-2(1H)-one	5.931083	266.0804	2818.0625	45325.57813	C9H13N3O5	Pyrimidine nucleosides	UHDGCWIWMRVCDJ-XVFCMESISA-N	O=C1N=C(N)C=CN1C2OC(CO)C(O)C2O
(9S,10S,17S)-3-(((2R,4S,5S,6R)-4,5-dihydroxy-6-methyltetrahydro-2H-pyran-2-yl)oxy)-5,14-dihydroxy-13-methyl-17-(5-oxo-2,5-dihydrofuran-3-yl)hexadecahydro-1H-cyclopenta[a]phenanthrene-10-	5.809583	557.2529	1212.6875	15560.0127	C29H42O9	Cardenolide glycosides and derivatives	QBILRDAMJUPXCX-QNCBPSDCSA-N	O=CC12CCC(OC3OC(C)C(O)C(O)C3)CC2(O)CCC4C1CCC5(C)C(C6=CC(=O)OC6)CCC45O
leonurine	5.971233	312.1566	2693.125	43797.15234	C14H21N3O5	Gallic acid and derivatives	WNGSUWLDMZFYNZ-UHFFFAOYSA-N	O=C(OCCCCNC(=N)N)C1=CC(OC)=C(O)C(OC)=C1
Norharman	9.889116	169.0628	2322.8125	20517.02148	C11H8N2	Beta carbolines	AIFRHYZBTHREPW-UHFFFAOYSA-N	N1C2=CC=CC=C2C2=CC=NC=C12
Uric acid	5.002117	167.0396	2281.5	28584.98242	C5H4N4O3	Xanthines	LEHOTFFKMJEONL-UHFFFAOYSA-N	OC1=NC2=C(N1)N=C(O)N=C2O
(6R,7R)-7-azaniumyl-3-methyl-8-oxo-5-thia-1-azabicyclo[4.2.0]oct-2-ene-2-	7.800817	215.0549	2234.1875	25891.81836	C8H10N2O3S	Alpha amino acids and derivatives	NVIAYEIXYQCDAN-CLZZGJSISA-N	O=C([O-])C1=C(C)CSC2N1C(=O)C2[NH3+]
Loganin	4.5668	435.1508	4529.9375	58858.44531	C17H26O10	Iridoid O-glycosides	AMBQHHVBBHTQBF-UHFFFAOYNA-N	COC(=O)C1=COC(OC2OC(CO)C(O)C(O)C2O)C2C(C)C(O)CC12
SMZ-Pt	4.6463	427.1006	2496.5625	33466.875	C17H16N8O4S	NA	LVUVWJASQQPDSL-UHFFFAOYSA-N	CC1=CC(=NO1)NS(C2=CC=C(C=C2)NCC=3C=NC4=C(C(=NC(=N)N4)O)N3)(=O)=O
(E)-2-(hydroxymethyl)-3-(3-oxo-5-propan-2-yl-4,5,6,7-tetrahydro-1H-2-benzofuran-4-yl)prop-2-enoic acid	6.793684	279.1235	15871.6875	271541.0938	C15H20O5		YWASZTJGPFRWMW-BJMVGYQFSA-N	O=C(O)C(=CC1C=2C(=O)OCC2CCC1C(C)C)CO
(1R,2R,4S,7R,8S,12R)-7-(furan-3-yl)-1,8,12,17,17-pentamethyl-3,6,16-trioxapentacyclo[9.9.02,4.02,8.012,18]icos-13-ene-5,15,20-trione	9.798017	453.1916	8546.875	96771.58594	C26H30O7		MAYJEFRPIKEYBL-DKBZVKCESA-N	O=C1OC(C)(C)C2CC(=O)C3(C)C(CCC4(C)C(OC(=O)C5OC534)C6=COC=C6)C2(C=C1)C
(1S,2S)-2-(methylamino)-1-phenylpropan-1-ol hydrochloride	4.8473	202.0856	2536.8125	41217.27344	C10H16ClNO	Phenylpropanes	BALXUFOVQVENIU-KXNXZCPBSA-N	Cl.OC(C=1C=CC=CC1)C(NC)C
Madecassoside	6.833517	973.4642	6378	76687.75	C48H78O20	Triterpene saponins	BNMGUJRJUUDLHW-UHFFFAOYNA-N	CC1CCC2(CCC3(C)C(=CCC4C5(C)CC(O)C(O)C(C)(CO)C5C(O)CC34C)C2C1C)C(=O)OC1OC(COC2OC(CO)C(OC3OC(C)C(O)C(O)C3O)C(O)C2O)C(O)C(O)C1O
PURINE	6.528217	121.0636	9860	118192.4141	C5H4N4	Purines and purine derivatives	KDCGOANMDULRCW-UHFFFAOYSA-N	N=1C=NC=2N=CNC2C1
HYDROQUINIDINE	8.08295	325.2005	24632.9375	414188.0938	C20H26N2O2		LJOQGZACKSYWCH-UHFFFAOYSA-N	OC(C=1C=CN=C2C=CC(OC)=CC21)C3N4CCC(C3)C(C4)CC
D-(+)-Malic acid	1.493633	133.0159	41023.5625	950163.5625	C4H6O5	Beta hydroxy acids and derivatives	BJEPYKJPYRNKOW-UWTATZPHSA-N	O[C@H](CC(O)=O)C(O)=O
MMV045105	7.082517	322.1398	98722.1875	1168779.25	C19H19N3O2	Diphenylethers	WQWBZRVAFAMASC-UHFFFAOYSA-N	N=1NC(=CC1N2CCOCC2)C=3C=CC(OC=4C=CC=CC4)=CC3
1-hydroxy-10-methylacridone	5.687917	248.0816	12616.125	150617.2344	C14H11NO2	Acridones	NHYDBJFGZCQFEO-UHFFFAOYSA-N	O=C1C=2C=CC=CC2N(C=3C=CC=C(O)C13)C
2-((2H-pyrazolo[3,4-d]pyrimidin-4-yl)amino)ethanol	10.13807	202.0755	1769.5	17889.37695	C7H9N5O	Pyrazolo[3,4-d]pyrimidines	LCOTUPXIQXPIPA-UHFFFAOYSA-N	OCCNC1=NC=NC2=NNC=C21
N-Methylnicotinamide	5.204783	137.0589	4764	76010.59375	C7H8N2O	Nicotinamides	ZYVXHFWBYUDDBM-UHFFFAOYSA-N	CN=C(c1cccnc1)O
Gastrodin	5.931083	309.0964	8924.5	149477.0625	C13H18O7	Phenolic glycosides	PUQSUZTXKPLAPR-UJPOAAIJSA-N	OCC1=CC=C(OC2OC(CO)C(O)C(O)C2O)C=C1
(2R,3R,4S,5R)-2-(6-amino-9H-purin-9-yl)-5-(hydroxymethyl)tetrahydrofuran-3,4-diol	6.40905	290.0905	8454.5	97661.85938	C10H13N5O4	Purine nucleosides	OIRDTQYFTABQOQ-KQYNXXCUSA-N	OCC1OC(N2C=NC=3C(=NC=NC32)N)C(O)C1O
N-(1-oxo-1-(3,5,6,7-tetrahydrospiro[imidazo[4,5-c]pyridine-4,4'-piperidin]-1'-yl)propan-2-yl)furan-2-	7.28	380.1835	1399.4375	16601.11523	C18H23N5O3	N-acyl-alpha amino acids and derivatives	JQBOIGWHYPDAGZ-UHFFFAOYSA-N	O=C(NC(C(=O)N1CCC2(NCCC=3N=CNC32)CC1)C)C=4OC=CC4
6-methoxy-7-(3-methylbut-2-enoxy)chromen-2-one	2.605567	259.0694	2354.5625	55718.55078	C15H16O4	Coumarins and derivatives	ZLPLFUBVEZVYDX-UHFFFAOYSA-N	O=C1OC=2C=C(OCC=C(C)C)C(OC)=CC2C=C1
haematein	6.369383	323.0559	5070.0625	90878.4375	C16H12O6	1-benzopyrans	HLUCICHZHWJHLL-INIZCTEOSA-N	O=C1C=C2C(C=C1O)=C3C=4C=CC(O)=C(O)C4OCC3(O)C2
Pristimerin	9.759	487.2654	1785.5625	14537.18555	C30H40O4	Triterpenoids	JFACETXYABVHFD-WXPPGMDDSA-N	O=C1C=C2C(=CC=C3C2(C)CCC4(C)C5CC(C(=O)OC)(C)CCC5(C)CCC34C)C(=C1O)C
Pyrimethamine	10.73048	249.0894	2660.5	22891.41016	C12H13ClN4	Aminopyrimidines and derivatives	WKSAUQYGYAYLPV-UHFFFAOYSA-N	CCC1=NC(=N)NC(N)=C1C1=CC=C(Cl)C=C1
5,8-dimethoxyquinoline-2-carbaldehyde	4.688817	218.0735	1545.0625	25638.14453	C12H11NO3	Quinolines and derivatives	JTUCCIJVUMWRPQ-UHFFFAOYSA-N	O=CC1=NC2=C(OC)C=CC(OC)=C2C=C1
Cyclo-prolylglycine	9.50885	155.0834	1475.3125	28390.13867	C7H10N2O2	Alpha amino acids and derivatives	OWOHLURDBZHNGG-YFKPBYRVSA-N	OC1=NCC(=O)N2CCC[C@@H]12
Thalsimine	1.883467	637.2707	5888.125	80382.28125	C38H40N2O7	Tannins	YWNUNVSMOKMJMG-LJAQVGFWSA-N	COC1=C2OC3=CC=C(CC4=NCCC5=CC(OC)=C(OC6=C(OC)C(OC)=C(OC)C7=C6[C@H](CC(C=C1)=C2)N(C)CC7)C=C45)C=C3
DL-threo-beta-Methylaspartic	6.448717	148.0725	2241.375	26605.41016	C5H9NO4	L-alpha-amino acids	LXRUAYBIUSUULX-UHFFFAOYNA-N	CC(C(N)C(O)=O)C(O)=O
Monocrotaline	6.647867	326.1696	2976.3125	45085.66797	C16H23NO6	Pyrrolizines	QVCMHGGNRFRMAD-XFGHUUIASA-N	O=C1OC2CCN3CC=C(COC(=O)C(O)(C)C(O)(C)C1C)C32
3-(2,4-dihydroxyphenyl)-5-hydroxy-8,8-dimethyl-6-(3-methylbut-2-enyl)pyrano[2,3-	2.171933	419.1518	3021.125	87883.76563	C25H24O6		ZSSVYEUXLNVQMH-UHFFFAOYSA-N	O=C1C(=COC=2C=3C=CC(OC3C(=C(O)C12)CC=C(C)C)(C)C)C=4C=CC(O)=CC4O
Secoisolariciresinol diglucoside	7.484317	685.2642	3858.375	54599.32031	C32H46O16	Lignan glycosides	SBVBJPHMDABKJV-UHFFFAOYNA-N	COC1=C(O)C=CC(CC(COC2OC(CO)C(O)C(O)C2O)C(COC2OC(CO)C(O)C(O)C2O)CC2=CC(OC)=C(O)C=C2)=C1

4,7,8-trimethoxy-3,5-dimethylchromen-2-one	8.81325	265.119	2223.625	39663.42969	C14H16O5	Coumarins and derivatives	PKKTXAMCHLIVDS-UHFFFAOYSA-N	O=C1OC2=C(OC)C(OC)=CC(=C2C(OC)=C1C)C
[3,4,5-tris(acetyloxy)-6-[(5-hydroxy-1,4-dioxo-3-{[3,4,5-tris(acetyloxy)-6-[(acetyloxy)methyl]oxan-2-yl]oxy}-1,4-dihydronaphthalen-2-yl)oxy]oxan-2-yl]methyl acetate	1.279333	867.2314	4520.0625	64333.33984	C38H42O23	Naphthoquinones	COICYYHTLYZMEX-UHFFFAOYSA-N	O=C(OCC1OC(OC=2C(=O)C=3C=CC=C(O)C3C(=O)C2OC4OC(COC(=O)C)C(OC(=O)C)C(OC(=O)C)C4OC(=O)C)C(OC(=O)C)C(OC(=O)C)C1OC(=O)C)C
Pesticide2_Methabenzthiazuron_C10H11N3OS_1-(1,3-Benzothiazol-2-yl)-1,3-	4.7283	222.0654	11117.25	167922.9375	C10H11N3OS	Benzothiazoles	RRVIAQKBTUQODI-UHFFFAOYSA-N	OC(=NC)N(C1=NC=2C=CC=CC2S1)C
Norboldine	6.8067	336.1002	10789.625	321192.6875	C18H19NO4	Aporphines	KYVJVURXKAZJRK-LBPRGKRZSA-N	OC1=CC2=C(C=C1OC)C3=C(OC)C(O)=CC4=C3C(NCC4)C2
((6aR,9R,10aR)-7-methyl-4,6,6a,7,8,9,10,10a-octahydroindolo[4,3-fg]quinolin-9-yl)methanol	10.73048	279.1348	11095.125	92893.96875	C16H20N2O	Clavines and derivatives	UFKTZIXVYHGAES-WDBKCZKBSA-N	OCC1CN(C)C2CC3=CNC4=CC=CC(=C43)C2C1
(2R,3R)-3,7-dihydroxy-6-methoxy-2-phenyl-2,3-dihydrochromen-4-one	2.963217	285.0954	2063	67790.5	C16H14O5		GUMFPOIJOXLCLA-JKSUJKDBSA-N	O=C1C2=CC(OC)=C(O)C=C2OC(C=3C=CC=CC3)C1O
N,O-dimethylcoclaurine	5.204783	314.1735	16522.6875	212758.3906	C19H23NO3		DLDZDBDYUZLISX-KRWDZBQOSA-N	OC=1C=C2C(=CC1OC)CCN(C)C2CC3=CC=C(OC)C=C3
6-(2-aminopyrimidin-4-yl)-4-butyl-7-hydroxy-8-methyl-2H-chromen-2-one	5.931083	326.1357	3896.875	73082.95313	C18H19N3O3	7-hydroxycoumarins	KMPDNLYICWJCPY-UHFFFAOYSA-N	O=C1OC2=C(C=C(C=3N=C(N=CC3)N)C(O)=C2C)C(=C1)CCCC
Alliin	7.800817	200.0315	2369.125	26559.73438	C6H11NO3S	L-alpha-amino acids	XUHLIQGRKRUKPH-JDLXGLENSA-N	O=C(O)C(N)CS(=O)CC=C
9-Anthracenecarboxylic acid	6.63535	221.0802	5544.75	65302.29297	C15H10O2	Anthracenecarboxylic acids	XGWFJBFNAQHLEF-UHFFFAOYSA-N	C=1C=CC=2C(C1)=CC3=CC=CC=C3C2C(O)=O
Ginsenoside Rf	10.35782	799.4813	1773.25	21983.23828	C42H72O14	Triterpenoids	UZIOUZHBUYLDHW-UHFFFAOYNA-N	CC(C)=CCCC(C)(O)C1CCC2(C)C1C(O)CC1C3(C)CCC(O)C(C)(C)C3C(CC21C)OC1OC(CO)C(O)C(O)C1OC1OC(CO)C(O)C(O)C1O
Benzoic acid + 2O, O-Hex	1.736133	315.0705	138262.25	2174113.75	C13H16O9	Phenolic glycosides	CBTFERBMQQAROP-UHFFFAOYNA-N	O=C(O)C2=CC(OC1OC(CO)C(O)C(O)C1(O))=CC=C2(O)
6-ethyl-3-(4-isopropoxyphenyl)-4-oxo-4H-chromen-7-yl acetate	8.733267	367.1399	1573.6875	17310.875	C22H22O5	Isoflavones	LPVOPZMNIAIRJY-UHFFFAOYSA-N	O=C(OC1=CC=2OC=C(C(=O)C2C=C1CC)C=3C=CC(OC(C)C)=CC3)C
[6,10a-dihydroxy-4-(hydroxymethyl)-4,7,11b-trimethyl-9-oxo-1,2,3,4a,5,6,6a,7,11,11a-decahydronaphtho[2,1-	6.3987	407.2086	7822.9375	153399	C22H32O7		GWEGFCVAOPNYPT-UHFFFAOYSA-N	O=C1OC2(O)C(=C1)C(C)C3C(O)C(OC(=O)C)C4C(C)(CO)CCCC4(C)C3C2
Metronidazole	4.8868	172.0758	1430.875	21782.89648	C6H9N3O3	Nitroimidazoles	VAOCPAMSLUNLGC-UHFFFAOYSA-N	CC1=NC=C(N1CCO)N(=O)=O
Circumdatin F_130023	6.8067	292.0992	533758.8125	8748740	C17H13N3O2	Pyrimidodiazepines	QMACPZZZSHLKJM-JTQLQIEISA-N	O=C1NC(C2=NC=3C=CC=CC3C(=O)N2C=4C=CC=CC14)C
maltotriose	1.295317	503.1587	9105.5625	142608	C18H32O16		FYGDTMLNYKFZSV-PXXRMHSHSA-N	OCC1OC(OC2C(O)C(O)C(OC2CO)OC3C(O)C(O)C(O)OC3CO)C(O)C(O)C1O
(+)-bicuculline	6.329883	368.1115	3628.8125	40994.73047	C20H17NO6	Phthalide isoquinolines	IYGYMKDQCDOMRE-ZWKOTPCHSA-N	O=C1OC(C2=CC=C3OCOC3=C12)C4C5=CC=6OCOC6C=C5CCN4C
alpha-D-Galactose-1-phosphate	4.6863	259.0263	2921.625	45040.06641	C6H13O9P	Monosaccharide phosphates	HXXFSFRBOHSIMQ-FPRJBGLDSA-N	OC[C@H]1O[C@H](OP(O)(O)=O)[C@H](O)[C@@H](O)[C@H]1O
methyl 2-((4-methyl-2-oxo-2H-chromen-7-yl)oxy)propanoate	6.8067	263.0908	4130.8125	88518.76563	C14H14O5	Coumarins and derivatives	NPTOZJLCWXEGKK-UHFFFAOYSA-N	O=C1OC=2C=C(OC(C(=O)OC)C)C=CC2C(=C1)C
FA 18:4+2O	8.60075	307.1904	40670.1875	627591.25	C18H28O4	Oxidized fatty acids	KLFMLBSZQZVKDC-UHFFFAOYNA-N	O=C(O)CCCCCCCC(=O)C=CC=CC=CC(O)CC
Apigenin-6-C-glucoside-7-O-glucoside	2.883233	593.1486	1368.6875	43022.35938	C27H30O15	Flavonoid-7-O-glycosides	HGUVPEBGCAVWID-UHFFFAOYNA-N	OCC1OC(OC2=C(C3OC(CO)C(O)C(O)C3O)C(O)=C3C(=O)C=C(OC3=C2)C2=CC=C(O)C=C2)C(O)C(O)C1O
Ophiopogonoside A	6.0904	441.2421	1142.1875	21169.70313	C21H38O8	Terpene glycosides	DQRUOTCFENUXKV-UHFFFAOYSA-N	OCC1OC(OC2C(CCC3(C)C(O)CCC(O)(C)C23)C(C)C)C(O)C(O)C1O
Ethyl caffeate	4.688817	209.0931	4586.6875	76626.34375	C11H12O4	Coumaric acids and derivatives	WDKYDMULARNCIS-GQCTYLIASA-N	O=C(OCC)C=CC1=CC=C(O)C(O)=C1
Gemcitabine	4.7283	264.0742	1402.8125	23531.75977	C9H11F2N3O4	Pyrimidine 2'-deoxyribonucleosides	SDUQYLNIPVEERB-QPPQHZFASA-N	OC[C@H]1O[C@@H](N2C=CC(=N)N=C2O)C(F)(F)[C@@H]1O
Gentiopicrin	6.250067	379.103	2396.5625	26678.99414	C16H20O9	O-glycosyl compounds	DUAGQYUORDTXOR-GPQRQXLASA-N	O=C1OCC=C2C1=COC(OC3OC(CO)C(O)C(O)C3O)C2C=C
Methyl gallate	8.203783	185.0449	1914.1875	22919.78711	C8H8O5	Galloyl esters	FBSFWRHWHYMIOG-UHFFFAOYSA-N	O=C(OC)C1=CC(O)=C(O)C(O)=C1
PYRIDOXAMINE	9.54855	169.0995	2456.8125	28004.44727	C8H12N2O2	Pyridoxamine 5'-phosphates	NHZMQXZHNVQTQA-UHFFFAOYSA-N	OC=1C(=NC=C(C1CN)CO)C
4-hydroxycoumarin	5.850584	163.0599	18269.625	221469.1094	C9H6O3	4-hydroxycoumarins	VXIXUWQIVKSKSA-UHFFFAOYSA-N	O=C1OC=2C=CC=CC2C(O)=C1
Kainic Acid	6.448717	236.0806	6795.1875	83102.05469	C10H15NO4	Kainoids	VLSMHEGGTFMBBZ-OOZYFLPDSA-N	O=C(O)CC1C(NCC1C(=C)C)C(=O)O
Harmine hydrochloride	8.5329	249.0876	1221.625	22801.46289	C13H13ClN2O	Harmala alkaloids	VNPLYCKZIUTKJM-UHFFFAOYSA-N	Cl.N=1C=CC=2C=3C=CC(OC)=CC3NC2C1C
Coptisine	7.3595	321.0975	44655.375	593604.875	C19H14NO4+	Protoberberine alkaloids and derivatives	XYHOBCMEDLZUMP-UHFFFAOYSA-N	O1C=2C=CC3=CC=4C5=CC=6OCOC6C=C5CC[N+]4C=C3C2OC1
5,6,7-Trimethoxycoumarin	10.77032	237.0897	2254.5	19415.68555	C12H12O5		FOBNRKTURPWTQX-UHFFFAOYSA-N	O=C1OC2=CC(OC)=C(OC)C(OC)=C2C=C1
(2R,7S,13R,14R,16S,19R,20S)-19-(furan-3-yl)-11-hydroxy-9,9,13,20-tetramethyl-4,8,15,18-tetraoxahexacyclo[11.9.0.0?,?.0?,??.0??,??.0??,??]docos-10-ene-5,12,17-trione	8.640583	483.1613	11469.25	139568.7344	C26H28O9	Steroid lactones	SNGHLUWTFLYPMT-UBTUHEMNSA-N	O=C1OCC23C(=C(O)C(=O)C4(C)C2CCC5(C)C(OC(=O)C6OC645)C7=COC=C7)C(OC3C1)(C)C
4-Coumaroylcholine	5.085134	250.1405	4089.25	51857.65625	C14H20NO3	Coumaric acid esters	WRLCNNSWESNISL-UHFFFAOYSA-O	C[N+](C)(C)CCOC(=O)C=CC1=CC=C(O)C=C1
Silymarin 10.03 min	5.555583	481.1002	4383.6875	62857.27344	C25H22O10	Flavonolignans	SEBFKMXJBCUCAI-WAABAYLZSA-N	COC1=CC(=CC=C1O)[C@@H]1OC2=CC(=CC=C2O[C@H]1CO)[C@H]1OC2=CC(O)=CC(O)=C2C(=O)[C@@H]1O
2',5'-Dihydroxy-4-	2.883233	269.0682	4476.9375	172996.6563	C16H14O4		FUQGIYDZGLORRC-XBXARRHUSA-N	O=C(C=CC1=CC=C(OC)C=C1)C2=CC(O)=CC=C2O
3-[(2S,3R,4S,5S,6R)-4,5-dihydroxy-6-(hydroxymethyl)-3-[(2S,3R,4S,5S,6R)-3,4,5-trihydroxy-6-(hydroxymethyl)oxan-2-yl]oxyoxan-2-yl]oxy-2-(3,4-dihydroxyphenyl)-5-hydroxy-7-	1.695783	639.1518	1812.4375	36270.57813	C28H32O17		CEZKIFXYWPTANH-HGVYTWBRSA-N	O=C1C(OC2OC(CO)C(O)C(O)C2OC3OC(CO)C(O)C(O)C3O)=C(OC4=CC(OC)=CC(O)=C14)C=5C=CC(O)=C(O)C5
Ketamine	4.688817	238.0931	2000.9375	33202.77734	C13H16ClNO	Chlorobenzenes	YQEZLKZALYSWHR-UHFFFAOYNA-N	CNC1(CCCCC1=O)C1=CC=CC=C1Cl
5,7-dimethoxy-2-phenyl-4H-chromen-4-one	2.611933	283.117	25465.5625	523368.625	C17H14O4	7-O-methylated flavonoids	JRFZSUMZAUHNSL-UHFFFAOYSA-N	O=C1C=C(OC2=CC(OC)=CC(OC)=C12)C=3C=CC=CC3
Dehydroeffusol	6.448717	251.0946	6359.1875	83033.67969	C17H14O2	Phenanthrols	GSSPKCPIRDPBQE-UHFFFAOYSA-N	OC=1C=C(C(=C)C=2C(=CC=C3C2C=CC(O)=C3C)C1
(R,E)-6-acetyl-7,9-dihydroxy-2-(1-((3-(3-(2-methoxyethyl)-1,2,4-oxadiazol-5-yl)propyl)amino)ethylidene)-8,9b-dimethyldibenzo[b,d]furan-1,3(2H,9bH)-dione	6.5682	534.2112	1269.0625	22404.2793	C26H29N3O8	Acetophenones	GCKXCHKHBLNGAF-WGKDYUSESA-N	O=C1C=C2OC3=C(C(=O)C)C(O)=C(C(O)=C3C2(C(=O)C1=C(NCCCC4=NC(=NO4)CCOC)C)C)C
L-Oxonoreleagnine	6.964517	187.103	1194.5625	19072.76758	C11H10N2O	Beta carbolines	FZHZQHNKCPJTNQ-UHFFFAOYSA-N	OC1=NCCC2=C1NC1=CC=CC=C21
5-Methoxypsoralen	1.4143	215.0323	44408.25	638375.4375	C12H8O4	5-methoxypsoralens	BGEBZHIAGXMEMV-UHFFFAOYSA-N	O=C1OC=2C=C3OC=CC3=C(OC)C2C=C1

7-hydroxy-2-(4-hydroxy-3,5-dimethoxyphenyl)-5-[(2S,3R,4S,5S,6R)-3,4,5-trihydroxy-6-(hydroxymethyl)oxan-2-	2.605567	491.1148	4200.75	88535.92188	C23H24O12		FLSOTPIEFVBPBU-LDBVRRDLSA-N	O=C1C=C(OC2=CC(O)=CC(OC3OC(CO)C(O)C(O)C3O)=C12)C=4C=C(OC)C(O)=C(OC)C4
curvularin	8.4026	291.1228	9749	111597.2188	C16H20O5	Macrolides and analogues	VDUIGYAPSXCJFC-UHFFFAOYSA-N	O=C1OC(C)CCCCCC(=O)C=2C(O)=CC(O)=CC2C1
Soyasaponin Bb	9.71885	941.51	1965.6875	21832	C48H78O18	Triterpene saponins	PTDAHAWQAGSZDD-UHFFFAOYNA-N	CC1OC(OC2C(O)C(O)C(CO)OC2OC2C(O)C(O)C(OC2OC2CCC3(C)C(CCC4(C)C3CC=C3C5CC(C)(C)CC(O)C5(C)CCC43C)C2(C)CO)C(O)=O)C(O)C(O)C1O
7-ethenyl-1,4a,7-trimethyl-3,4,6,8,8a,9,10,10a-octahydro-2H-phenanthrene-1-carboxylic	7.197	301.2015	5161.5	79784.08594	C20H30O2	Diterpenoids	TVHDZSRRHQKNEZ-UHFFFAOYSA-N	O=C(O)C1(C)CCCC2(C3=CCC(C=C)(C)CC3CCC12)C
Saikosaponin C	7.963783	925.4822	2544.8125	42673.89844	C48H78O17	Triterpene saponins	PYJMYPPFWASOJX-UHFFFAOYNA-N	CC1OC(OC2C(COC3OC(CO)C(O)C(O)C3O)OC(OC3CCC4(C)C(CCC5(C)C4C=CC4=C6CC(C)(C)CCC6(CO)C(O)CC54C)C3(C)C)C(O)C2O)C(O)C(O)C1O
Triamterene	2.651917	254.115	2394.1875	54395.73828	C12H11N7			n1c(nc(N)c2nc(c(nc12)N)c3ccccc3)N
9,13-Dimethyl-17-oxo-14-(5-oxo-2,5-dihydro-3-furanyl)tetracyclo[11.3.1.01,10.04,9]heptadec-6-yl ?-D-glucopyranosyl-(1->6)-?-D-glucopyranosyl-(1->4)-2,6-dideoxy-3-O-methyl-D-lyxo-	7.279167	839.4017	1509.5	24877.08789	C42H64O17		YUXPMUIKXCUNQY-MCRXNTOMSA-N	O=C1OCC(=C1)C2CCC34C(=O)C2(C)CCC3C5(C)CCC(OC6OC(C)C(OC7OC(COC8OC(CO)C(O)C(O)C8O)C(O)C(O)C7O)C(OC)C6)CC5CC4
Hypaphorine	5.204783	269.1156	16148.6875	208761.5313	C14H18N2O2	L-alpha-amino acids	AOHCBEAZXHZMOR-UHFFFAOYSA-N	O=C([O-])C(CC1=CNC=2C=CC=CC21)[N+](C)(C)C
Proline	5.324616	116.0603	1456.5625	33122.07422	C5H9NO2	Proline and derivatives	ONIBWKKTOPOVIA-UHFFFAOYNA-N	OC(=O)C1CCCN1
(2R)-6-methylpiperidine-2-carboxylic acid	7.598817	166.0642	1319.125	20305.51563	C7H13NO2	L-alpha-amino acids	IHLDCUQUFBWSJU-PRJDIBJQSA-N	O=C(O)C1NC(C)CCC1
Lycopene	7.881467	559.4141	2159.3125	23591.5625	C40H56	Carotenes	OAIJSZIZWZSQBC-GYZMGTAESA-N	C(=CC=C(C=CC=C(C=CC=C(C)CCC=C(C)C)C)C)C=C(C=CC=C(C=CC=C(C)CCC=C(C)C)C)C
4-Acetyl-2-methoxyphenyl 6-O-(6-deoxy-alpha-L-mannopyranosyl)-beta-D-	2.368917	519.1743	3185.5625	65149.29688	C21H30O12	Phenolic glycosides	ZUXLIBRTWRBKLQ-NUASCYGXSA-N	O=C(C1=CC=C(OC2OC(COC3OC(C)C(O)C(O)C3O)C(O)C(O)C2O)C(OC)=C1)C
Scutellarioside II	6.556366	507.1482	67507.8125	784417.375	C24H28O12		MVTLXFDHKDVAIC-LQQBYVAQSA-N	O=C(OCC12OC2C(O)C3C=COC(OC4OC(CO)C(O)C(O)C4O)C31)C=CC5=CC=C(O)C=
Nitrazepam	4.7283	282.0867	15350.875	239089.8438	C15H11N3O3	1,4-benzodiazepines	KJONHKAYOJNZEC-UHFFFAOYSA-N	O=N(=O)C=1C=CC=2N=C(O)CN=C(C=3C=CC=CC3)C2C1
(9E)-11a-hydroxy-3,6,10-trimethyl-6,7,8,11-tetrahydro-4H-cyclodeca[b]furan-2,5-dione	7.116333	263.1255	16153.1875	289660.2188	C15H20O4	Germacranolides and derivatives	SCOXWKWLFRIELY-WEVVVXLNSA-N	O=C1OC2(O)C(=C1C)CC(=O)C(C)CCC=C(C)C2
[4-[4,5-dihydroxy-6-methyl-3-(3,4,5-trihydroxyoxan-2-yl)oxyoxan-2-yl]oxy-6-[2-(3,4-dihydroxyphenyl)ethoxy]-5-hydroxy-2-(hydroxymethyl)oxan-3-yl] (E)-3-(3,4-	1.4143	755.2429	5929.4375	96100.57813	C34H44O19		UDHCHDJLZGYDDM-QPJJXVBHSA-N	O=C(OC1C(OC(OCCC2=CC=C(O)C(O)=C2)C(O)C1OC3OC(C)C(O)C(O)C3OC4OCC(O)C(O)C4O)CO)C=CC5=CC=C(O)C(O)=C5
Isorhamnetin 3-galactoside	6.71435	477.1195	6722.875	83997.42188	C22H22O12		CQLRUIIRRZYHHS-UHFFFAOYSA-N	O=C1C(OC2OC(CO)C(O)C(O)C2O)=C(OC=3C=C(O)C=C(O)C13)C=4C=CC(O)=C(OC)
Bufalin	6.754017	431.2275	8967.625	192756.5156	C24H34O4	Bufanolides and derivatives	QEEBRPGZBVVINN-UHFFFAOYNA-N	CC12CCC3C(CCC4CC(O)CCC34C)C1(O)CCC2C1=COC(=O)C=C1
Lucidenic acid D	7.399	515.278	1316.6875	36932.99609	C29H38O8	Triterpenoids	LTJSBYAKDOGXLX-JTJCPSTFSA-N	O=C(OC1C(=O)C2=C(C(=O)CC3C(C(=O)CCC23C)(C)C)C4(C(=O)CC(C(C)CCC(=O)O)C14C)C)C
2-(hydroxymethyl)-4(3H)-quinazolinone	4.052516	177.0762	180576.0625	9569617	C9H8N2O2	Quinazolines	QXGGDTNBLBJSIJ-UHFFFAOYSA-N	O=C1N=C(NC=2C=CC=CC12)CO
3-(4-hydroxyphenyl)-1-phenyl-2-propen-1-one	6.170233	247.0731	3098	43384.25781	C15H12O2	Retrochalcones	PWWCDTYUYPOAIU-DHZHZOJOSA-N	O=C(C=CC1=CC=C(O)C=C1)C=2C=CC=CC2
(-)-Podophyllotoxin	1.453967	459.1323	17095.4375	254090.9063	C22H22O8	Podophyllotoxins	YJGVMLPVUAXIQN-UHFFFAOYNA-N	COC1=CC(=CC(OC)=C1OC)C1C2C(COC2=O)C(O)C2=CC3=C(OCO3)C=C12
Coumaroyl Hexoside (isomer of 690, 692)	5.199266	325.0937	6333.4375	90095.57031	C15H18O8	Coumaric acid and derivatives	DSNCQKUYZOSARM-UHFFFAOYNA-N	O=C(OC1OC(CO)C(O)C(O)C1(O))C=CC2=CC=C(O)C=C2
neoeriocitrin	5.51575	595.1556	1442	25882.25195	C27H32O15		OBKKEZLIABHSGY-DOYQYKRZSA-N	O=C1C2=C(O)C=C(OC3OC(CO)C(O)C(O)C3OC4OC(C)C(O)C(O)C4O)C=C2OC(C5=CC=C(O)C(O)=C5)C1
Irbesartan	8.922234	427.232	20119.875	320292.25	C25H28N6O	Biphenyls and derivatives	YOSHYTLCDANDAN-UHFFFAOYSA-N	CCCCC1=NC2(CCCC2)C(=O)N1CC1=CC=C(C=C1)C1=CC=CC=C1C1=NNN=N1
2-hydroxy-4-methyl-3H-benzo[e][1,4]diazepin-5(4H)-one	3.1049	191.056	1656.1875	37501.52344	C10H10N2O2	1,4-benzodiazepines	ZVHUQIMPAVOVTR-UHFFFAOYSA-N	O=C1C=2C=CC=CC2N=C(O)CN1C
(2S,3R,4S,5S,6R)-2-[(2R,3R,4S,5S,6R)-4,5-dihydroxy-6-(hydroxymethyl)-2-(3-hydroxy-6-methylheptoxy)oxan-3-yl]oxy-6-	8.4026	515.215	18024.1875	226644.4375	C20H38O12	Fatty acyl glycosides of mono- and disaccharides	OCLJUQSRRLIINU-CBMLQZBESA-N	OCC1OC(OC2C(O)C(O)C(OC2OCCC(O)CCC(C)C)CO)C(O)C(O)C1O
Syringic acid	4.7283	221.0565	2839.9375	47359.09766	C9H10O5	Gallic acid and derivatives	JMSVCTWVEWCHDZ-UHFFFAOYSA-N	O=C(O)C1=CC(OC)=C(O)C(OC)=C1
quinine	6.528217	325.1832	1423.3125	19272.94922	C20H24N2O2	Cinchona alkaloids	LOUPRKONTZGTKE-VOMFEXJBSA-N	OC(C=1C=CN=C2C=CC(OC)=CC21)C3N4CCC(C3)C(C=C)C4
5-(hydroxymethyl)pyrimidine-2,4-diol	5.204783	143.0481	9582.25	126316.3125	C5H6N2O3	Pyrimidones	JDBGXEHEIRGOBU-UHFFFAOYSA-N	OC1=NC=C(C(O)=N1)CO
Thymidine??5'-monophosphate	5.51575	321.0615	2406.75	29760.26172	C10H15N2O8P	Pyrimidine 2'-deoxyribonucleoside monophosphates	GYOZYWVXFNDGLU-UHFFFAOYNA-N	O=C1N=C(O)C(=CN1C2OC(COP(=O)(O)O)C(O)C2)C
methyl (2S,3R,4S)-3-ethenyl-4-[2-(3,4,5-trihydroxybenzoyl)oxyethyl]-2-[3,4,5-trihydroxy-6-(hydroxymethyl)oxan-2-yl]oxy-	5.08095	541.1494	3799.5	44045.04688	C24H30O14		SMTKSCGLXONVGL-FMDXMRPMSA-N	O=C(OC)C1=COC(OC2OC(CO)C(O)C(O)C2O)C(C=C)C1CCOC(=O)C3=CC(O)=C(O)C(O)=C3
N-Methylparoxetine	5.647083	344.1822	6122.5	75000.95313	C20H22FNO3	Phenylpiperidines	MOJZPKOBKCXNKG-UHFFFAOYNA-N	FC1=CC=C(C=C1)C2CCN(C)CC2COC3=CC=C4OCOC4=C3
skullcapflavone II	5.880567	373.092	8582.9375	117311.6641	C19H18O8		GMQFOKBGMKVUQZ-UHFFFAOYSA-N	O=C1C=C(OC2=C(OC)C(OC)=C(OC)C(O)=C12)C=3C(O)=CC=CC3OC
Dihydropalmatine	1.596317	354.1538	2695.4375	35748.65234	C21H23NO4	Protoberberine alkaloids and derivatives	PTPHDVKWAYIFRX-UHFFFAOYSA-N	O(C=1C=CC=2C=C3C4=CC(OC)=C(OC)C=C4CCN3CC2C1OC)C
C10H11NO2	3.755867	178.0922	4473.25	145985.5781	C10H11NO2	Formula predicted		
1-(2-(1H-indol-3-	6.448717	220.084	2877.125	40350.75781	C11H13N3S	3-alkylindoles	HRBGONPRSGPFMT-UHFFFAOYSA-N	S=C(N)NCCC1=CNC=2C=CC=CC21
3,4-Dihydroxymandelate	2.8434	183.0296	3606.25	70838.65625	C8H8O5	Catechols	RGHMISIYKIHAJW-UHFFFAOYNA-N	OC(C(O)=O)C1=CC(O)=C(O)C=C1
1-Cinnamoylpyrrolidine	4.8868	224.1044	1306.8125	20441.75781	C13H15NO	Cinnamic acids and derivatives	JSIGICUAXLIURX-CMDGGOBGSA-N	O=C(C=CC=1C=CC=CC1)N2CCCC2
5-(3,4-dihydroxyphenyl)-6,7-dimethyl-5,6,7,8-tetrahydronaphthalene-2,3-diol	6.48855	283.131	1721.375	21646.21094	C18H20O4	Aryltetralin lignans	MUBLYIKZGKHTMF-UHFFFAOYSA-N	OC1=CC=C(C=C1O)C2C3=CC(O)=C(O)C=C3CC(C)C2C
Sulfadimethoxine	5.555583	309.0599	24913.125	375375.4063	C12H14N4O4S	Aminobenzenesulfonamides	ZZORFUFYDOWNEF-UHFFFAOYSA-N	COC1=NC(OC)=NC(NS(=O)(=O)C2=CC=C(N)C=C2)=C1
Enterolactone	9.5612	297.111	8089.75	107956.2969	C18H18O4	Dibenzylbutyrolactone lignans	HVDGDHBAMCBBLR-UHFFFAOYNA-N	OC1=CC=CC(CC2COC(=O)C2CC2=CC(O)=CC=C2)=C1
10-Azabenzo[a]pyrene	5.204783	254.0902	1902.75	28212.10156	C19H11N	Pyrenes	BYFIUFNRSNKODH-UHFFFAOYSA-N	C1=CC=2C=CC3=CC=4C=CC=NC4C=5C=CC(=C1)C2C35

PFCA-perfluoroalkyl_Hsubstituted_1DB	1.775783	256.9735	1368.9375	17074.43945	C6H2F8O2	PFSA	CMFAKTRZLATJOA-UPHRSURJSA-N	O=C(O)C=C(F)C(F)(F)C(F)(F)C(F)(F)F
5-hydroxy-2,2,6,6-tetramethyl-4-[2-methyl-1-[2,4,6-trihydroxy-3-(2-methylpropanoyl)phenyl]propyl]c	8.122617	431.2244	4973.25	78571.46875	C24H32O7		JUXUXBIHGDQKND-UHFFFAOYSA-N	O=C(C1=C(O)C=C(O)C(=C1O)C(C=2C(=O)C(C(=O)C(C2O)(C)C)(C)C)C(C)C)C(C)C
gibberellic acid	5.396767	345.1519	29227.1875	487529.6875	C19H22O6		IXORZMNAPKEEDV-OBDJNFEBSA-N	O=C(O)C1C2C3(OC(=O)C2(C)C(O)C=C3)C4CCC5(O)C(=C)CC14C5
Quinic acid	1.374633	191.0564	92107.1875	1552754.75	C7H12O6	Quinic acids and derivatives	AAWZDTNXLSGCEK-TUNDHVGDSA-N	O=C(O)C1(O)CC(O)C(O)C(O)C1
Threonine	1.555817	120.0794	2142.875	41968.45703	C4H9NO3	L-alpha-amino acids	AYFVYJQAPQTCCC-UHFFFAOYNA-N	CC(O)C(N)C(O)=O
Hydroxypyridin + C5H10NO or 6-Hydroxypseudooxynicotine (not validated)	9.54855	195.1143	5960.6875	55497.38281	C10H14N2O2	Nicotine and derivatives	UMLOUOBDBGOHHR-UHFFFAOYSA-N	O=C(C=1C=NC(O)=CC=1)CCCNC
3-Methoxycinnamic acid	4.688817	179.0845	3565.125	64814.46875	C10H10O3	Coumaric acids and derivatives	LZPNXAULYJPXEH-AATRIKPKSA-N	COC1=CC=CC(\C=C\C(O)=O)=C1
Dimethenamide ESA	8.853416	322.0697	1541.4375	17084.37305	C12H19NO5S2	Heteroaromatic compounds	YMYKMSAZEZQEER-UHFFFAOYNA-N	COCC(C)N(C(=O)CS(O)(=O)=O)C1=C(C)SC=C1C
Oxaprozin	6.40905	294.1126	44573.25	556643.3125	C18H15NO3	Phenyl-1,3-oxazoles	OFPXSFXSNFPTHF-UHFFFAOYSA-N	OC(=O)CCC1=NC(=C(O1)C1=CC=CC=C1)C1=CC=CC=C1
zearalenol	8.882067	319.1516	15944.625	273524.9375	C18H24O5		FPQFYIAXQDXNOR-XVNBXDOJSA-N	O=C1OC(C)CCCC(O)CCCC=CC=2C=C(O)C=C(O)C12
Monuron	5.357267	197.0447	13473.125	169366.5781	C9H11ClN2O	N-phenylureas	BMLIZLVNXIYGCK-UHFFFAOYSA-N	CN(C)C(=O)NC1=CC=C(Cl)C=C1
khellin	9.889116	261.0909	1346.5	11917.41016	C14H12O5	Furanochromones	HSMPDPBYAYSOBC-UHFFFAOYSA-N	O=C1C=C(OC=2C(OC)=C3OC=CC3=C(OC)C12)C
C20H34O9	7.7648	417.2081	3128.75	61068.02344	C20H34O9	Formula predicted		
2-(2-hydroxy-6-methylhept-5-en-2-yl)-7-(3-methylbut-2-enyl)-2,3-dihydro-1-benzofuran-5-carboxylic acid	8.282766	357.2041	4179.125	72895.49219	C22H30O4		GZLIPAFSJXROEC-UHFFFAOYSA-N	O=C(O)C=1C=C(C=2OC(CC2C1)C(O)(C)CCC=C(C)C)CC=C(C)C
